# Pervaporation Membranes Based on Polyelectrolyte Complex of Sodium Alginate/Polyethyleneimine Modified with Graphene Oxide for Ethanol Dehydration

**DOI:** 10.3390/polym16091206

**Published:** 2024-04-25

**Authors:** Mariia Dmitrenko, Olga Mikhailovskaya, Roman Dubovenko, Anna Kuzminova, Danila Myznikov, Anton Mazur, Konstantin Semenov, Yury Rusalev, Alexander Soldatov, Sergey Ermakov, Anastasia Penkova

**Affiliations:** 1 St. Petersburg State University, 7/9 Universitetskaya nab., St. Petersburg 199034, Russia; st113220@student.spbu.ru (O.M.); r.dubovenko@spbu.ru (R.D.); a.kuzminova@spbu.ru (A.K.); dan.myznikov@gmail.com (D.M.); a.mazur@spbu.ru (A.M.); s.ermakov@spbu.ru (S.E.); 2 Pavlov First Saint Petersburg State Medical University, L’va Tolstogo ulitsa 6–8, St. Petersburg 197022, Russia; semenov1986@yandex.ru; 3 The Smart Materials Research Institute, Southern Federal University, 178/24 Sladkova St., Rostov-on-Don 344090, Russia; yuri.rusalev@gmail.com (Y.R.); soldatov@sfedu.ru (A.S.)

**Keywords:** sodium alginate, graphene oxide, polyethyleneimine, polyelectrolyte complex, pervaporation, ethanol dehydration

## Abstract

Pervaporation is considered the most promising technology for dehydration of bioalcohols, attracting increasing attention as a renewable energy source. In this regard, the development of stable and effective membranes is required. In this study, highly efficient membranes for the enhanced pervaporation dehydration of ethanol were developed by modification of sodium alginate (SA) with a polyethylenimine (PEI) forming polyelectrolyte complex (PEC) and graphene oxide (GO). The effect of modifications with GO or/and PEI on the structure, physicochemical, and transport characteristics of dense membranes was studied. The formation of a PEC by ionic cross-linking and its interaction with GO led to changes in membrane structure, confirmed by spectroscopic and microscopic methods. The physicochemical properties of membranes were investigated by a thermogravimetric analysis, a differential scanning calorimetry, and measurements of contact angles. The theoretical consideration using computational methods showed favorable hydrogen bonding interactions between GO, PEI, and water, which caused improved membrane performance. To increase permeability, supported membranes without treatment and cross-linked were developed by the deposition of a thin dense layer from the optimal PEC/GO (2.5%) composite onto a developed porous substrate from polyacrylonitrile. The cross-linked supported membrane demonstrated more than two times increased permeation flux, higher selectivity (above 99.7 wt.% water in the permeate) and stability for separating diluted mixtures compared to the dense pristine SA membrane.

## 1. Introduction

The development of sustainable processes is receiving increasing attention worldwide because of environmental concerns, global climate change, and the growth of the population [1]. Membrane technologies are related to sustainable processes due to their advantages (environmental friendliness, economic advantage, low energy consumption, etc.) [2,3]. The most promising and actively developing membrane method for separating liquid mixtures of low molecular weight substances is pervaporation [4]. The term “pervaporation” was originally a combination of the words “permselective” and “evaporation” [5]. During pervaporation, components of the feed in contact with a dense membrane selectively penetrate the membrane, and they are collected in the vapor phase on the reverse side. The advantage of pervaporation is that the separation is not based on thermodynamic equilibrium between the vapor and liquid phases (as in distillation) [4]. The permeate concentration is determined by the permeability of substances through the membrane, which depends on the diffusion rate and solubility in the membrane [6]. This makes pervaporation an alternative technology to traditional separation methods (for example, distillation). Energy losses in distillation are very high due to the presence of azeotropes or components with close boiling points [6]. Thus, pervaporation is a less energy-intensive technology [6]. Pervaporation is also actively used and developed for the dehydration and recovery of solvents [7], which are important operations for industry and laboratories [8].

Pervaporation can also be considered and applied most effectively among membrane methods for the purification, concentration, and fractionation of liquid biofuels [9]. Fossil fuel reserves are currently being depleted. Biofuels, among renewable energy sources, can fill in as a superior choice to lessen the dependence on fossil fuels. In particular, bioalcohols as liquid biofuels produced by microbial fermentation require intensive purification and concentration for high product purity [10]. To obtain purified ethanol from a binary ethanol–water mixture, two energy-intensive separation steps are required: (1) the distillation to 92.4–94 wt.% ethanol, and (2) the dehydration of ethanol to obtain anhydrous ethanol (an ethanol concentration of above an azeotropic composition with water) by pressure, extractive, azeotropic distillation, etc. Another environmental problem is the distillation residue, namely a vinasse of large quantities (from 1 L of ethanol to about 15 L of vinasse) [10]. The application of pervaporation may solve this problem. With the correct selection of membranes, this makes it possible to reduce energy costs, the labor intensity of the operation, and achieve high separation efficiency.

For dehydration purposes, membranes from hydrophilic polymers such as chitosan (CS), polyvinyl alcohol, sodium alginate (SA), cellulose derivatives, polyamide, etc., are actively used [11]. In recent years, polyelectrolytes have received considerable attention as a promising membrane material for liquid separation due to their selective water permeation [12]. Membranes from a polyelectrolyte complex (PEC) exhibit optimal functionality due to the combination of polyelectrolyte properties and the “salting-out-effect” towards organic substances [13,14]. Multilayer membranes are also now being developed using various polyelectrolytes such as CS, alginic acid, polyacrylic acid, polyethyleneimine (PEI), polydiallyldimethyl ammonium chloride, etc. This work is focused on the production of pervaporation membranes based on a PEC by the mixing of eco-friendly polyelectrolytes, such as anionic biopolymer SA and cationic PEI which have not received great attention until now.

SA as a natural polysaccharide obtained from algae is actively used in the food, biomedical, textile, and cosmetic industries due to its advantages such as environmental friendliness, solubility in water, biocompatibility, and low cost [15,16,17]. It is also a progressive membrane material for fuel cells, nanofiltration, ultrafiltration, and especially for pervaporation [18,19,20,21]. SA membranes are characterized by insufficient permeability and low stability in dilute aqueous solutions. This is a significant drawback for their use in industry. Procedures such as grafting, cross-linking, blending, and the creation of mixed matrix and composite SA-based membranes have been carried out to improve performance [20]. In this work, the improvement of SA was carried out by introducing PEI for the creation of a PEC and graphene oxide (GO).

The creation of a PEC by adding PEI to SA would increase the permeability and stability of the pervaporation membrane in diluted solutions. It would do this due to hydrophilization of the matrix because of the PEC’s charged nature and ionic cross-linking between polyelectrolytes, respectively. However, it also would cause a decrease in selectivity to water due to the simultaneous penetration of components through the membrane because of the expansion of the free volume between the polymer chains [14]. To the best of our knowledge, there are only two works on creating pervaporation PEC membranes from SA and PEI. The work [22] was devoted to the development of pervaporation polyelectrolyte multilayer membranes from PEI and SA alternatively deposited onto an ultrafiltration hydrolyzed PAN substrate by a dynamic layer-by-layer technique. These membranes were developed for the separation of ethanol–water mixtures which consisted of inorganic or organic acid. The membrane formed from the 1.5 bilayer ((SA/PEI)_1.5_/PAN) exhibited optimal transport characteristics: a permeation flux of 1.203 kg/(m^2^ h) and a separation factor of 1542 in the pervaporation dehydration of ethanol (10 wt.% water) with a pH of 3 at 60 °C. Dense PEC membranes cross-linked with glutaraldehyde (from SA and PEI) were prepared for the pervaporation dehydration of alcohols (methanol (10 wt.% water), ethanol (4 wt.% water), isopropanol (4–13.5 wt.% water), and t-butanol (13.5 wt.% water)) in a work by Anjali Devi et al. [14]. The variation of PEI content in PEC membranes (from 10 to 40 wt.%) demonstrated that a PEC membrane containing 40 wt.% PEI was found to have optimum separation parameters: a permeation flux of 0.095 kg/(m^2^ h) and a separation factor of 1249 in the pervaporation separation of a 90 wt.% isopropanol–water mixture at 30 °C. However, PEC membrane stability was not studied for the pervaporation of diluted solutions in these works. They were tested in the pervaporation dehydration of alcohols with not more than 13.5 wt.% water in the feed.

Two-dimensional (2D) materials such as GO are attracting growing interest as modifiers in membrane separations for the dehydration of organics [20]. GO and its derivatives have been actively used to modify alginate-based membranes for pervaporation desalination [23,24] and dehydration of alcohols [25,26,27,28,29,30], where it showed potential for use. The modification of SA and PEC-based membranes with carbon nanoparticles of GO would significantly change the morphology of the polymer matrix, the surface hydrophilicity of a membrane, and the free volume between the chains of polymers. It would cause a significant improvement in transport characteristics (an increase in permeation flux and/or selectivity) of modified membranes in pervaporation [31]. Modification with GO leads to enhanced water sorption due to its polar nature and functional groups. Thus, GO particles in a membrane act as selective channels for water and as an absorber for ethanol [32], resulting in improved permeability [33,34]. It is also worth noting that GO interacts with groups of SA and PEI in the PEC composition, forming various bonds and linking polymer chains, leading to improved PEC-based membrane selectivity [26].

Thus, the aim of the present work was to study the effect of modifications of SA with GO and PEI which form a PEC on membrane characteristics and the development of highly efficient pervaporation membranes for the enhanced dehydration of ethanol in a wide concentration range (4–90 wt.% water). The introduction influence of different concentrations of GO into the SA membrane and the selection of the optimal concentration of the modifier in the polymer matrix were studied. Next, the effect of PEI content in the PEC composition on membrane characteristics was investigated. After this, two modifications were combined by the preparation of a PEC (from SA and PEI) membrane modified with optimal GO content. To improve further performance and for promising use in industry, untreated and cross-linked supported membranes were developed and studied. They consisted of a dense thin selective layer from the optimal PEC/GO composite deposited onto a developed porous substrate from polyacrylonitrile (PAN). Chemical cross-linking with glutaraldehyde (GA) of the supported PEC-based membranes allowed for the use of them for the separation of diluted solutions. GA, as a cross-linking agent, linked the polymer chains of SA and PEI by covalent bonding in PEC membranes, contributing to their stability. To explain changes, the structure and physicochemical properties of membranes were investigated by spectroscopic (FTIR and NMR) and microscopic (SEM, TEM, and AFM) methods, a thermogravimetric analysis (TGA), a differential scanning calorimetry, and measurements of contact angles. Theoretical considerations carried out using computational methods were presented to confirm the effect of modifications on membrane performance.

## 2. Materials and Methods

### 2.1. Materials

SA (Jiangsu Benefit Ocean Technology Co., LTD., Jiangsu, China) obtained from OOO “BIOPROD LLC” (St. Petersburg, Russia) and PEI (M.N. 60,000, 50 wt.% aq. solution, branched, ACROS ORGANICS, St. Petersburg, Russia) were used for the preparation of a PEC. GO was obtained from “Fullerene Technologies” (St. Petersburg, Russia) and used as a modifier for the modification of SA and PEC-based membranes. Polyacrylonitrile (PAN, Mw = 150,000 g/mol, Ming International Co., St. Petersburg, Russia) was applied for the preparation of a porous substrate for the development of supported membranes. N,N-dimethylacetamide (DMAc), GA, sulfuric acid (H_2_SO_4_), ethanol (EtOH), isopropanol (iPrOH) obtained from LLC “TD GALA-TRADE” and “Vekton” (St. Petersburg, Russia) were used without additional pretreatment.

### 2.2. Membrane Preparation

#### 2.2.1. Dense Membranes

The SA membranes were prepared as follows: SA was dissolved in water and stirred for 4 h at 45 °C to obtain a 1 wt.% polymer solution. The introduction of the modifier was carried out by the addition of GO dispersion (20 g/L) into the SA solution (1, 3, 5, and 7 wt.% of GO with respect to the polymer weight), stirring, and ultrasonic treating at an ambient temperature.

The basis for the preparation of a PEC for the manufacture of membranes was taken from the approach of mixing SA and PEI in different ratios from the work [14]. For PEC membrane preparation, 1 wt.% SA and 5 wt.% PEI solutions in water were mixed in different proportions to obtain the following polymer SA/PEI ratios: 90/10, 60/40, and 50/50 wt.%. The prepared solution was stirred for 1 h until homogeneity and treated with ultrasound. It should be noted that a further increase in PEI content in the PEC composition led to the loss of film-forming properties (namely, the inability to obtain a membrane with good mechanical properties) [14]. Firstly, the volume (bulk) modification was carried out by the addition of GO dispersion into the PEC solution (2.5 wt.% of GO with respect to the PEC (SA/PEI) weight or 5 wt.% with respect to the SA weight), as for the SA membranes.

**Figure 1:**
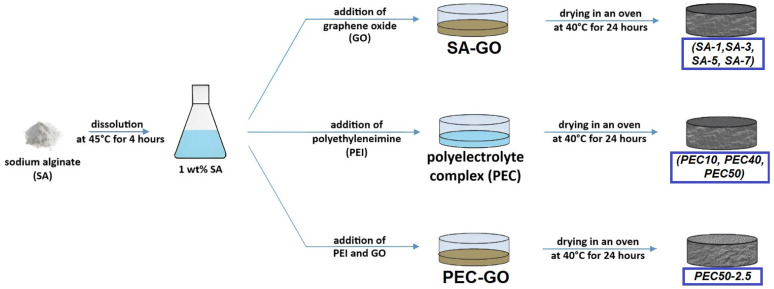
The preparation scheme of dense SA and PEC-based membranes.

Dense (nonporous) membranes were formed by solvent evaporation in an oven at 40 °C for 24 h of a solution (SA or PEC) or composite (SA/GO or PEC/GO) poured into a Petri dish (Figure 1). The obtained dense membranes had a thickness of 50 ± 5 μm measured by a micrometer.

The mechanism of PEC formation was as follows. The pH of the aqueous solution of PEI was 10.8, where the amino groups of PEI were not protonated [35]. The mixing of PEI, SA, and GO firstly occured at the molecular level. After film formation (drying), the first stage of interaction as ionic cross-linking between the SA, GO, and unprotonated –NH_2_ groups of PEI took place. Upon swelling, H+ diffused into the membrane and protonated the amino groups of PEI, running the second stage of in-situ ionic cross-linking between the protonated PEI, SA, and GO groups [36].

#### 2.2.2. Supported Membranes

**Figure 2:**
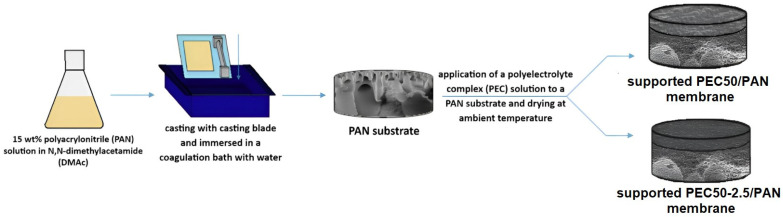
The preparation scheme of supported PEC-based membranes.

Supported membranes were developed by the deposition of a thin (nonporous) layer based on the PEC and PEC/GO (2.5%) composite onto the porous substrate (support) based on PAN (Figure 2), which was prepared using the phase inversion method (a non-solvent induced phase separation (NIPS) technique [37]). PAN was dissolved in DMAc to obtain a 15 wt.% solution. Then, it was deposited onto a glass support using a casting blade (200 µm gap width) and immersed in a coagulation water bath at an ambient temperature, where the polymer hardened and the porous structure of the substrate formed. To remove the remaining solvents from the structure, porous substrates were left in a water bath for 12 h at an ambient temperature.

The solutions for the preparation of a thin dense layer were prepared as for dense membranes. The thickness of the thin selective dense layer of supported membranes was 500 ± 100 nm, measured based on SEM micrographs.

#### 2.2.3. Cross-Linking of Membranes

To obtain membrane stability in diluted solutions, the supported PEC-based membranes were chemically cross-linked by immersion into an iPrOH–water (90/10 *v*/*v*) mixture containing 1 wt.% GA with 0.5 wt.% H_2_SO_4_ as the catalyst for 5 min [38]. After this, membranes were dried in an oven for 5 min at 40 °C.

The denotations of obtained membranes, their composition, and preparation conditions are presented in Table 1. The indications were as follows: the GO content (1, 2.5, 3, 5, and 7 wt.%) as a number, the PAN substrate through a slash for supported membranes, and the cross-linking agent GA as a superscript.

### 2.3. Pervaporation

**Figure 3:**
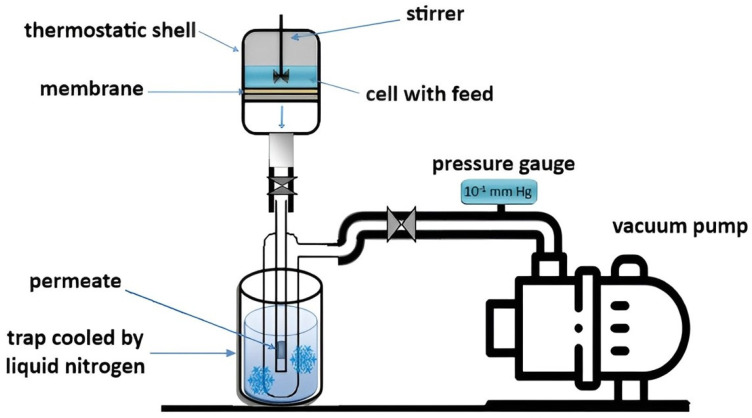
The pervaporation setup scheme.

The developed membranes were tested in a pervaporation separation of the EtOH–water (4–90 wt.%) mixture at an ambient temperature in a steady-state regime in cells with a 9.6 cm^2^ effective membrane area, with constant stirring (Figure 3). A feed of 100 g was utilized during experiments, and its composition was maintained constant to avoid irreproducibility of membrane transport parameters and to carry out pervaporation separation under the same conditions. The membranes were conditioned before starting the experiments; the first permeate sample within 30 min was removed from the analysis. During this time, the initial swelling and preliminary preconditioning of membranes (the second stage of in-situ ionic cross-linking between the protonated PEI, SA, and GO groups) in the feed were established. The permeate was collected for various periods of time (from 20 min to 5 h) depending on the permeation flux of membranes. It was important to obtain about 0.3 g of the permeate mass for the obtaining of reliable data on the permeate composition by gas chromatography. The determination of permeate and feed compositions was carried out using a gas chromatograph Chromatek Crystal 5000.2 (Chromatec, Nizhny Novgorod, Russia). It was equipped with a thermal conductivity detector and the “Hayesep R” column.

Each measurement was performed at least three times to obtain the average values used for subsequent analyses. The mean accuracy of the parameters was as follows: ±0.5% of permeate water content, ±5% of permeation flux for dense membranes, and ±10% of permeation flux for supported membranes.

The membrane permeation flux (*J*) was calculated as follows [39]:

(1) $$J=\frac{W}{A\cdot t},$$

where *A* is the membrane area (m^2^), *t* is the permeation time (h), and *W* is the weight of the permeated components (kg).

Based on data of component contents in the permeate and feed, the separation factor (β) was calculated as follows [40]:

(2) β=ywateryEtOHxwaterxEtOH,

where *x_water_* and *x_EtOH_* are fractions in the feed, and *y_water_* and *y_EtOH_* are fractions in the permeate.

To evaluate the effectiveness of membrane performance, the pervaporation separation index (*PSI*, kg/(m^2^h)) was calculated as follows:

(3) PSI=J·(β−1)

### 2.4. Fourier Transforms Infrared Spectroscopy (FTIR)

The structure of the developed SA and PEC-based membranes was studied by FTIR spectroscopy using an IRAffinity-1S spectrometer (Shimadzu, Kyoto, Japan) and an attenuated total reflectance (ATR) accessory (PIKE Technologies, Moscow, Russia) in the range of 450–4000 cm^−1^ at 25 °C.

### 2.5. Nuclear Magnetic Resonance Spectroscopy (NMR)

The membranes were investigated using a Bruker Avance III 400 WB NMR spectrometer (Bruker, Bremen, Germany). The magnetic field was set to 9.4 T with the CP/MAS probe of 4 mm. The MAS speed was 12.5 kHz for all experiments. To investigate ^13^C nuclei, a Larmor frequency of 100.64 MHz and tetramethylsilane (TMS) as an external reference were applied. For ^23^Na nuclei, a Larmor frequency of 105.87 MHz and liquid 1M∙D_2_O NaCl as an external reference were applied.

### 2.6. Scanning Electron Microscopy (SEM)

Membrane morphology (surface and cross-section, prepared by membrane submerging in liquid nitrogen and fracturing) was studied on a Zeiss Merlin SEM microscope (Carl Zeiss SMT, Oberkochen, Germany).

### 2.7. Transmission Electron Microscopy (TEM)

Using a microdispenser, the solutions were transferred to an ultra-thin carbon mesh on a copper frame (Lacey carbon mesh) for subsequent TEM research. The structures were studied using a Carl Zeiss Libra 200FE transmission electron microscope (Carl Zeiss SMT, Oberkochen, Germany) with an energy Ω filter and an accelerating voltage of 200 kV. The presented bright-field images were obtained in TEM mode using a CCD camera, and dark-field images were obtained in Scanning TEM (STEM) mode using a HAADF detector (E. A. Fischione Instruments Inc., Hanau, Germany).

### 2.8. Atomic Force Microscopy (AFM)

The surface topography of the SA and PEC-based membranes was studied by the AFM method using a NT-MDT NTegra Maximus atomic force microscope (NT-MDT Spectrum Instruments, Moscow, Russia) in tapping mode. The standard silicon cantilevers (15 N·m^−1^ rigidity) were applied.

### 2.9. Thermogravimetric Analysis (TGA)

The membrane thermochemical properties were investigated by the thermogravimetric analysis (TGA) method using a TG 209 F1 Libra thermobalance (Netzsch, Waldkraiburg, Germany) under an inert atmosphere Ar and a 10 °C/min heating speed.

### 2.10. Differential Scanning Calorimetry (DSC)

DSC thermograms were obtained on a DSC 204 F1 Phoenix (Netzsch, Waldkraiburg, Germany) in the temperature range of 30–210 °C at a 10 K/min heating rate.

### 2.11. Measurement of Contact Angle

The contact angle of water for the supported PEC-based membranes was measured by the sessile drop method with a Goniometer LK-1 instrument (NPK Open Science Ltd., Krasnogorsk, Russia). The software “DropShape” (the Laboratory of Mathematical Methods of Image Processing, Lomonosov Moscow State University, Moscow, Russia) was applied for contact angle analysis.

### 2.12. Computational Methods

To estimate thermodynamically stable conformations of model molecules by methods of molecular mechanics (MM2), ChemOffice CS Chem3D Ultra was used. Gaussian 16 W, Revision A.03 software software [41] was applied for the geometry optimization of structures, and VMD 1.9.3 software [42] were used for visualization. The optimization and characterization of molecular structures were carried out by Becke’s three-parameter Lee–Yang–Parr hybrid functional (B3LYP) [43,44,45] with a 6-311++G** basis set. The singlet state is the basic state of multiplicity observed in all systems. The geometry was optimized without symmetry restrictions. To establish the correct minima on the potential energy surface, Hessian matrices were calculated for all optimized model structures. Imaginary frequencies were not detected. The thermodynamic parameters were evaluated at 298.15 K and 1 atm. A multifunctional wavefunction analyzer (Multiwfn 3.7) [46] was used for bond order and Bader atoms-in-molecules topological analyses (QTAIM). VMD software (0.5 (e^1/3^ bohr)^−1^ isovalue, [–0.04; 0.02] e/bohr^3^ colorscale data range) was applied for 3D visualizations of the noncovalent interaction plot (NCIplot) [47].

The main results of quantum chemical calculations, such as Cartesian atomic coordinates of the optimized initial molecules and associates (Table S1), changes in thermodynamic potentials in the process of their formation (Tables S2 and S3), analyses of bond orders (Tables S4 and S5), the results of the topological analysis (Table S6), as well as NCIplots and QTAIM distribution of bond critical points and bond paths for the associates (Figures S1–S4) are presented in the Supplementary Materials.

## 3. Results

### 3.1. Investigation of Transport Properties

#### 3.1.1. Study of SA Membranes Modified with GO

**Figure 4:**
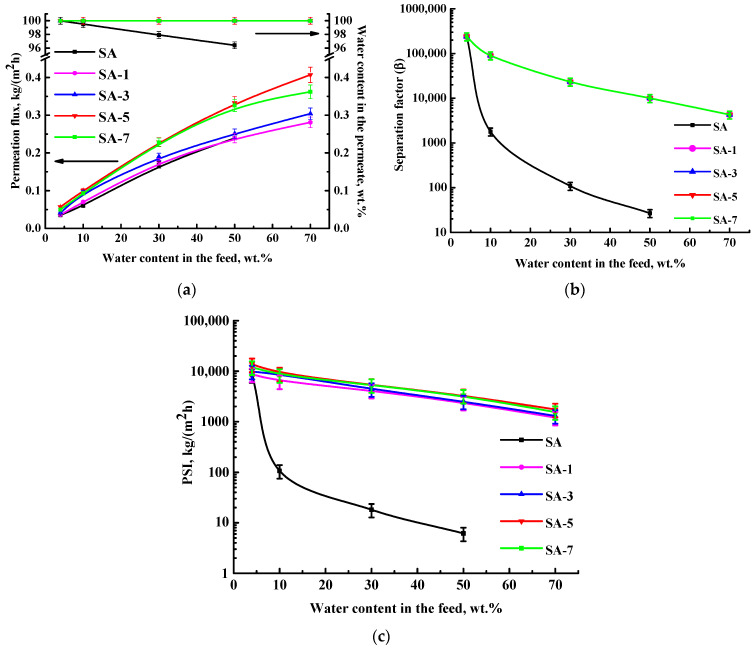
The dependence of (**a**) permeation flux, the water content in the permeate, (**b**) the separation factor, and (**c**) the PSI (in a log-scale) on water content in the feed for SA and SA/GO membranes in the pervaporation dehydration of ethanol (4–70 wt.% water). The water content in the permeate for all GO-modified membranes was 99.99 wt.%.

SA membranes modified with different GO concentrations (1, 3, 5, and 7 wt.%) were evaluated in the pervaporation ethanol dehydration in a wide concentration range (4–70 wt.% water) to assess the effect of GO modification and feed composition. Membrane performance (permeation flux, the water content in the permeate, the separation factor, and the PSI) is shown in Figure 4.

Pervaporation with up to 50 wt.% water in the feed was possible to carry out for the pristine SA membrane. The modification of SA with GO allowed for the use of modified membranes with up to 70 wt.% water in the feed. This confirms the GO cross-linking effect (confirmed by computational methods in the Supplementary Materials). The same effect was observed for membranes based on a SA-fullerenol composite in the pervaporation dehydration of iPrOH [48]. The increase in GO content from 1 to 7 wt.% in the SA matrix led to the rise of permeation flux (Figure 4a). Also, it should be noted that modified SA/GO membranes were highly selective with respect to water (99.99 wt.% in the permeate) compared to the pristine SA membrane. The SA-5 membrane (modified with 5 wt.% GO) had the highest permeation flux among all membranes and about 1.5 times improved permeation flux compared to the SA membrane. This may be due to hydrophilization of the SA-5 membrane surface because of the migration of functional oxygen-containing groups of GO [31] (a more energetically favorable hydrogen bonding interactions between the modifier GO and components of the feed compared to SA, Table S2 of the Supplementary Materials), and an increase in the roughness of the membrane structure (confirmed below by SEM and AFM data). The SA-7 membrane demonstrated the same high selectivity to water and decreased permeation flux compared to the SA-5 membrane due to the presence of agglomerates of carbon modifier (GO), causing an impermeable space for components to pass through the membrane [48]. In the work [33], it was confirmed that in a membrane prepared by filtration of GO suspensions on the cellulose ester substrate, the increase in GO agglomerates (flakes) led to a decrease in water flux maintaining the retention capacity of the membrane.

These changes (improved permeation flux and selectivity) for GO-modified membranes may be explained by the following factors. The modification with GO led to enhanced water sorption due to GO’s polar nature. Its functional groups led to improved hydrophilicity of the membrane surface, and GO particles acted as selective channels for water, resulting in improved permeability [33]. Also, GO provides interactions with the SA matrix (confirmed by NMR data below), due to which the free volume between the polymer chains decreased, leading to improved membrane selectivity The same effect was confirmed in the work [26]. It is also worth noting that the preferable interaction of GO with ethanol is observed by computational methods (Figure S2 of the Supplementary Materials), suggesting that ethanol adsorption with GO may occur, preventing its penetration through the membrane and maintaining high selectivity with increasing permeability of modified membranes. The improvement in ethanol adsorption upon modification of GO was also confirmed in the work [32].

To demonstrate the effectiveness of GO-modified membranes, the separation factor and the pervaporation separation index (PSI) were calculated based on obtained data of permeation flux and the water content in the permeate (Figure 4b,c). Separation factor values for all SA/GO membranes were equal due to the same selectivity (water content in the permeate) and higher compared to the SA membrane. The highest PSI was observed for the SA-5 membrane because of its highest permeability and selectivity. Thus, the developed SA-5 membrane modified by 5 wt.% GO has the optimal transport properties for the pervaporation dehydration of ethanol. However, for industrial applications, further enhancement of this membrane productivity is required. It can be achieved by a combination of modifications: the formation of a PEC by the addition of PEI into an SA matrix and the introduction of GO into it.

#### 3.1.2. Study of Membranes Based on PEC from SA/PEI

**Figure 5:**
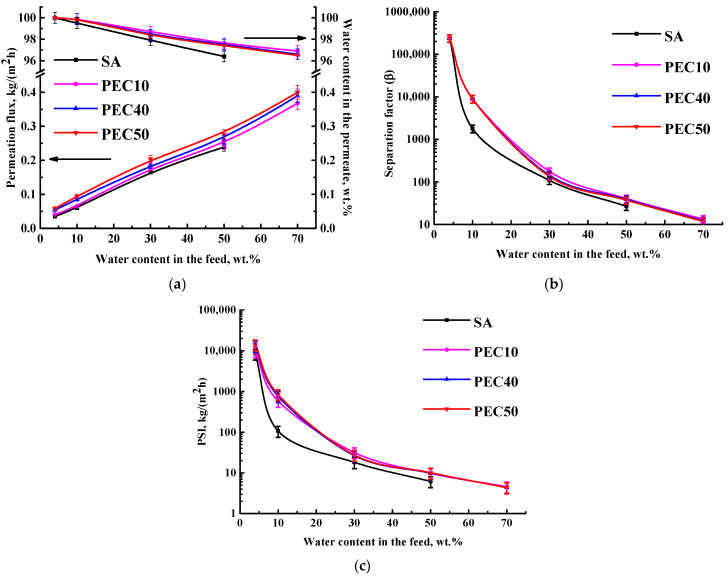
The dependence of (**a**) permeation flux, the water content in the permeate, (**b**) the separation factor, and (**c**) the PSI (in a log-scale) on water content in the feed for SA and PEC-based membranes in the pervaporation dehydration of ethanol (4–70 wt.% water).

To select optimal PEI content, an SA/PEI ratio (90/10, 60/40, 50/50) was varied in the PEC composition. PEC-based membranes were also studied in the pervaporation dehydration of ethanol. Their performance (permeation flux, the water content in the permeate, the separation factor, and the PSI) is presented in Figure 5. The SA membrane parameters were also repeated for comparison.

It was shown that the formation of the PEC (by adding PEI to the SA matrix) led to the PEC membrane stability separating until 70 wt.% water in the feed and a higher water content in the permeate (Figure 5a). It was due to ionic cross-linking between the NH_2_ group of PEI and –COONa group of SA [22] (confirmed below by FTIR data). The increase in PEI content in the PEC composition resulted in an increase in permeation flux and a slight reduction in the water content in the permeate for PEC membranes. This may be due to the fact that PEI, as a flexible polymer easily oriented in the presence of a liquid mixture, leads to the expansion of the free volume between the polymer chains [14], causing increased roughness of the inner structure and surface of the PEC membranes (confirmed below by SEM and AFM data). This provides improved permeability and reduced selectivity due to the simultaneous penetration of components. The separation factor and PSI, which is a quantitative indicator for comparing the performance of membranes, clearly show that the PEC50 membrane (from a 50/50 ratio of SA/PEI) has optimal transport characteristics (the highest permeation flux at a high level of selectivity).

**Figure 6:**
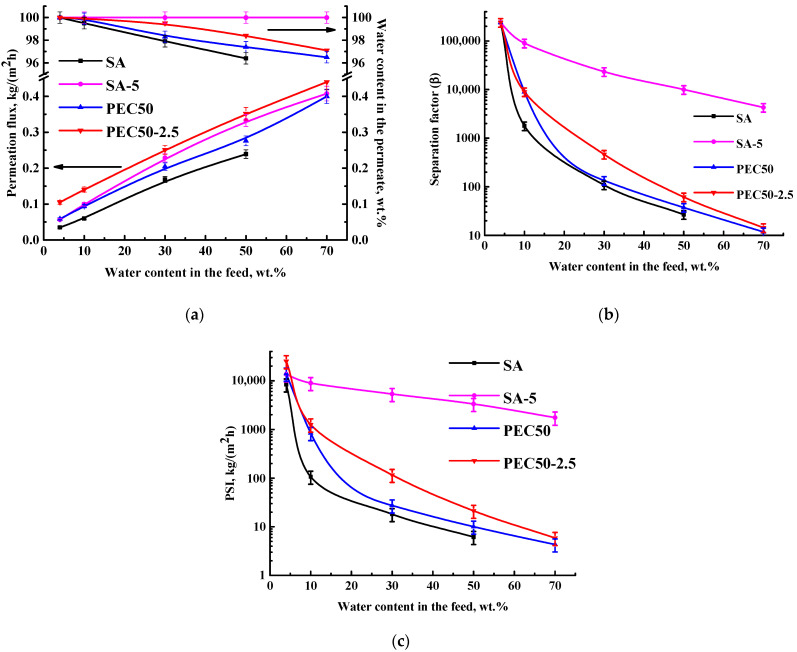
The dependence of (**a**) permeation flux, the water content in the permeate, (**b**) the separation factor, and (**c**) the PSI (in a log-scale) on water content in the feed for SA and PEC-based membranes, modified with GO in the pervaporation dehydration of ethanol (4–70 wt.% water).

To further improve the properties, this PEC50 membrane was modified with GO by the introduction of 2.5 wt.% GO with respect to the PEC weight or 5 wt.% relative to the SA weight, which was previously shown to be the optimal modifier concentration to improve the SA membrane performance. The PEC50-2.5 membrane performance (the permeation flux, the water content in the permeate, the separation factor, and the PSI) is presented in Figure 6. The parameters for the SA, PEC50, and SA-5 membranes are also demonstrated for comparison.

The combination of modifications (the formation of the PEC from SA/PEI and the introduction of GO into the PEC) led to a more than 1.5 times increased permeation flux and selectivity (water content in the permeate) for the PEC50-2.5 membrane compared to the pristine SA membrane (Figure 6a). The separation factor and PSI of the PEC50-2.5 membrane were lower compared to the SA-5 membrane. However, the PEC50-2.5 membrane had a higher PSI compared to the SA-5 membrane and athree times increased permeation flux compared to the pristine SA membrane in the pervaporation dehydration of an azeotropic EtOH–water mixture. It is a significant improvement, since azeotropes are difficult to separate by traditional methods (such as distillation). It usually requires the addition of carcinogenic entrainers [14]. Thus, this dense PEC50-2.5 membrane demonstrated optimal performance: the highest permeation flux and above 97 wt.% water in the permeate. For prospective industrial applications, the parameters of this membrane could be improved by the development of a supported membrane.

#### 3.1.3. Development and Study of Supported PEC-Based Membranes

**Figure 7:**
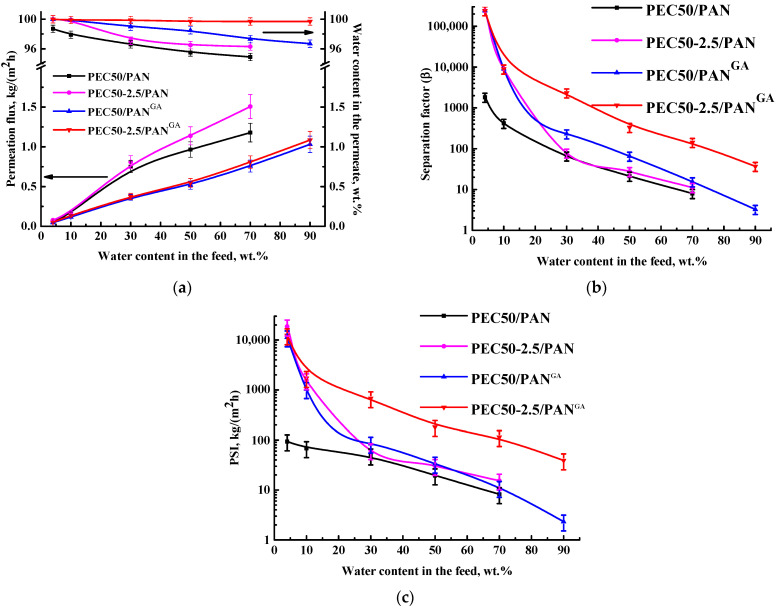
The dependence of (**a**) permeation flux, the water content in the permeate, (**b**) the separation factor, and (**c**) the PSI (in a log-scale) on water content in the feed for the supported untreated and cross-linked PEC-based membranes in the pervaporation dehydration of ethanol (4–90 wt.% water).

The improved performance of the PEC50-2.5 membrane was carried out by the creation of a supported membrane, his being the deposition of a thin dense selective layer from the PEC (50/50 ratio of SA/PEI), modified with 2.5 wt.% GO, onto the porous PAN substrate, which did not affect the mass transfer of components and ensured the good mechanical strength of the membrane. Also, to improve the stability of membranes in diluted mixtures, membranes were cross-linked with GA, which allowed them to be used across the entire concentration range of EtOH–water separation and allowed for improved selectivity. Transport properties of supported untreated PEC50-2.5/PAN and cross-linked PEC50-2.5/PAN^GA^ membranes are presented in Figure 7. The data for unmodified PEC50/PAN and PEC50/PAN^GA^ membranes are also shown for comparison.

The creation of supported membranes (PEC50/PAN and PEC50-2.5/PAN) and a decrease in the selective dense layer to 500 nm (1% of dense membrane thickness) made it possible to increase the permeation flux by more than three times for the dehydration of ethanol with above 30 wt.% water in the feed, maintaining the same level of selectivity (water content in the permeate) compared to dense membranes (PEC50 and PEC50-2.5, Figure 6a). The permeation flux of these supported membranes for the separation of mixtures containing 4 and 10 wt.% water was the same or slightly higher compared to the dense membranes due to the low water content in the feed. Some deviations from the theoretically calculated permeation flux of supported membranes compared to dense membranes may occur due to the influence of the substrate and defects in the thin dense layer during the preparation and separation process. The nature and porosity of the substrate may influence mass transfer through the membrane (namely, the diffusion path through the selective layer), as well as the pressure drop in the substrate layer on the driving force, resulting in a change in effectiveness of the selective dense layer [49,50]. The creation of a thin dense selective layer on a substrate may cause some defects, such as non-uniform thickness of the layer and some clogging of the substrate pores [49]. The influence of the choice of the nature and structure of substrates on transport properties was also previously confirmed for supported membranes with a thin dense selective layer based on carboxymethylcellulose, polyvinyl alcohol, CS, and SA [51,52,53,54].

The cross-linking of PEC50/PAN and PEC50-2.5/PAN membranes with GA (PEC50/PAN^GA^ and PEC50-2.5/PAN^GA^) led to the stability of cross-linked membranes during the pervaporation dehydration of ethanol until 90 wt.% water in the feed. These cross-linked membranes had improved selectivity (higher water content in the permeate) and decreased permeation flux compared to untreated supported membranes. This may be due to the reduction of the free volume between polymer chains [14] because of the cross-linking effect (confirmed below by FTIR and NMR data), resulting in the formation of a smoother and more even inner structure (confirmed below by SEM data) and decreased surface roughness of membranes (confirmed below by AFM data). The cross-linked GO-modified PEC50-2.5/PAN^GA^ membrane had the highest separation factor among all membranes (Figure 7b) and a higher selectivity (above 99.7 wt.% water in the permeate) compared to the unmodified supported PEC50/PAN^GA^ membrane, which may be caused by surface hydrophilization during the modification of GO (confirmed below by contact angle data). This PEC50-2.5/PAN^GA^ membrane is inferior to the untreated PEC50-2.5/PAN membrane in PSI value due to its lower permeability (Figure 7c).

**Figure 8:**
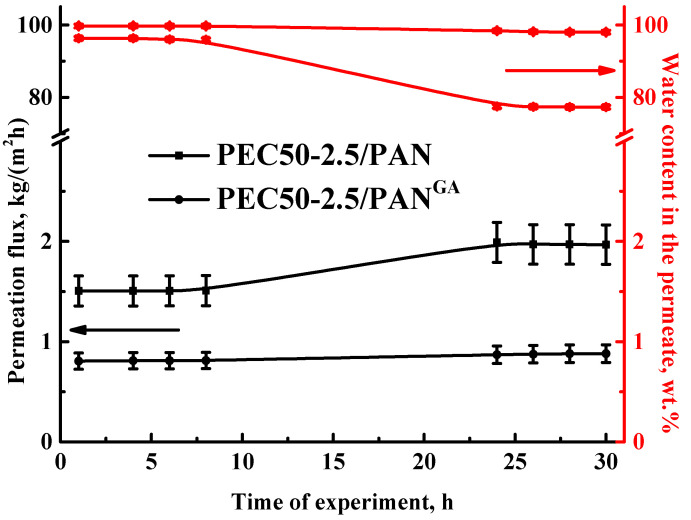
The dependence of permeation flux and water content in the permeate on experiment time for the supported untreated PEC50-2.5/PAN and cross-linked PEC50-2.5/PAN^GA^ membranes in the pervaporation dehydration of ethanol (70 wt.% water).

To evaluate the long-term stability of PEC-based membranes in high water content solutions, the supported untreated PEC50-2.5/PAN and cross-linked PEC50-2.5/PAN^GA^ membranes were tested in the pervaporation dehydration of ethanol (70 wt.% water) for 30 h (Figure 8). The experiment was carried out as follows: points were taken every two hours during the working day. Then, the membrane was left in the installation with the feed overnight, and the next day the points continued to be taken every two hours.

It was demonstrated that the permeation flux of the supported untreated PEC50-2.5/PAN membrane increased 1.3 times after 30 h of the pervaporation experiment, while the water content decreased from 96.3 to 77.3 wt.% in the permeate. These changes may be explained by the intense swelling of the untreated membrane in the feed, which leads to an increase in the free volume between the polymer chains, causing an increase in permeability and a decrease in selectivity due to the joint penetration of the components [55]. The cross-linking of this membrane (the cross-linked PEC50-2.5/PAN^GA^ membrane) led to more stable transport parameters: 1% increased permeation flux and decreased water content from 99.7 to 98 wt.% in the permeate after 30 h of the pervaporation dehydration of ethanol (70 wt.% water). This confirmed the stability of the developed cross-linked membrane.

Thus, the cross-linked supported membrane with a thin dense selective layer from the PEC (50/50 ratio of SA/PEI) modified with 2.5 wt.% GO deposited onto the PAN substrate (PEC50-2.5/PAN^GA^) had the optimal performance in the pervaporation dehydration of ethanol (4–90 wt.% water). More than two times increased permeation flux, higher selectivity (above 99.7 wt.% water in the permeate) and stability for separating diluted mixtures compared to the dense SA membrane were observed. The application of this membrane will be promising for industrial dehydration purposes.

### 3.2. Membrane Characterization

#### 3.2.1. Investigation of Membranes by Spectroscopic Methods

**Figure 9:**
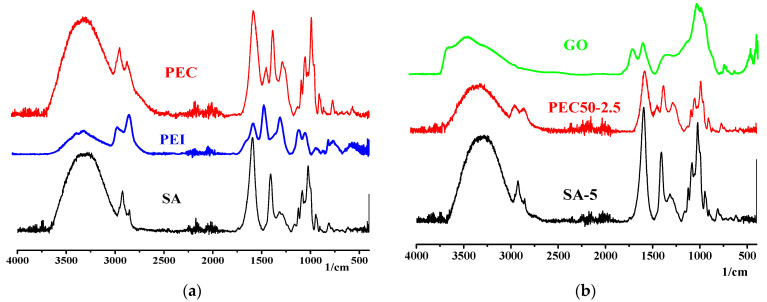
The FTIR spectra for (**a**) PEI, SA and PEC50 membranes, (**b**) GO, SA-5 and PEC50-2.5 membranes, modified with GO.

The structure of prepared dense SA and PEC-based membranes was studied by spectroscopic (FTIR and NMR) and microscopic (SEM, TEM and AFM) methods. FTIR spectra of membranes are presented in Figure 9.

**Figure 10:**
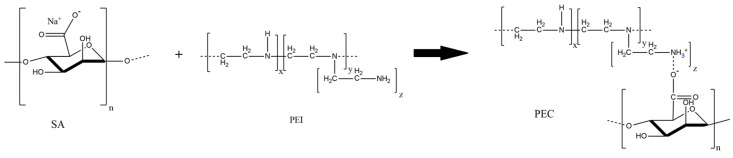
The scheme of ionic cross-linking between SA and PEI.

The FTIR spectrum of SA membrane shows the absorption bands at 3295 cm^−1^ (the stretching of the hydroxyl group), 1594 and 1408 cm^−1^ (asymmetric and symmetric stretching of the carboxylate salt group), 1315 (C–O), 1124 (C–C), 1084 (C–O), 1024 (C–O–O), and 938 cm^−1^ (C–O) corresponding to the saccharide structure [14,56,57,58,59]. The FTIR spectrum of PEI demonstrates its characteristic peaks at 1643 and 1573 cm^−1^, related to primary and secondary amines [14]. The formation of the PEC from SA/PEI led to the changes of absorption bands in the range of 1200–1700 cm^−1^: the peak shift was at 1594 to 1601 cm^−1^, and the appearance of peaks at 1471 (attributed to the protonation of –NH_2_ groups of PEI) and 1288 cm^−1^ (related to carbonyl stretching) [14,22]. The scheme of ionic cross-linking between SA (–COONa) and PEI (NH_3_^+^C) is presented in Figure 10.

The characteristic peaks of GO at 3401 (–OH stretching of carboxylic groups), 1724 (C=O stretching of carboxylic groups), and 1622 cm^−1^ (C=C stretching of the aromatic domain of graphene) were observed [44,45]. The modification of SA and the PEC with GO did not significantly change FTIR spectra of modified membranes; there was a reduction in intensity and shift of peaks from 3295 to 3289 cm^−1^ for the SA-5 membrane and from 3290 to 3282 cm^−1^ of the PEC50-2.5 membrane, and an increase in the intensity of peaks at 1600 cm^−1^ for the PEC50 membrane. This may be due to the overlap of characteristic peaks of the modifier and matrices, and may indicate weak interactions between GO and polymers [31].

**Figure 11:**
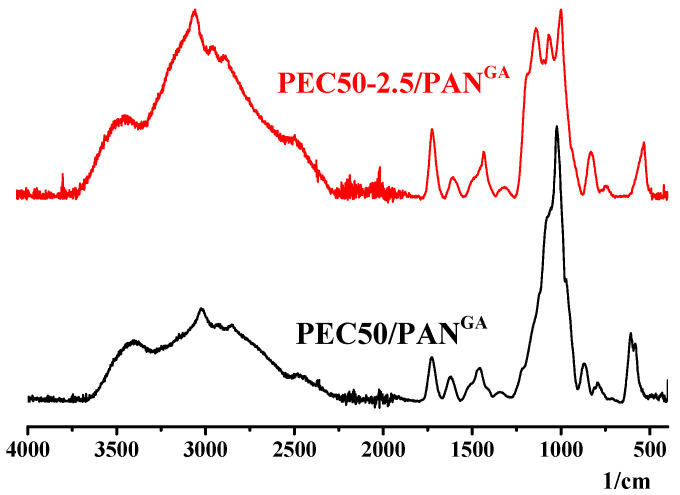
The FTIR spectra for supported untreated PEC50-2.5/PAN and cross-linked PEC50-2.5/PAN^GA^ membranes.

FTIR spectra of supported membranes (PEC50-2.5/PAN and cross-linked PEC50-2.5/PAN^GA^) are presented in Figure 11.

**Figure 12:**
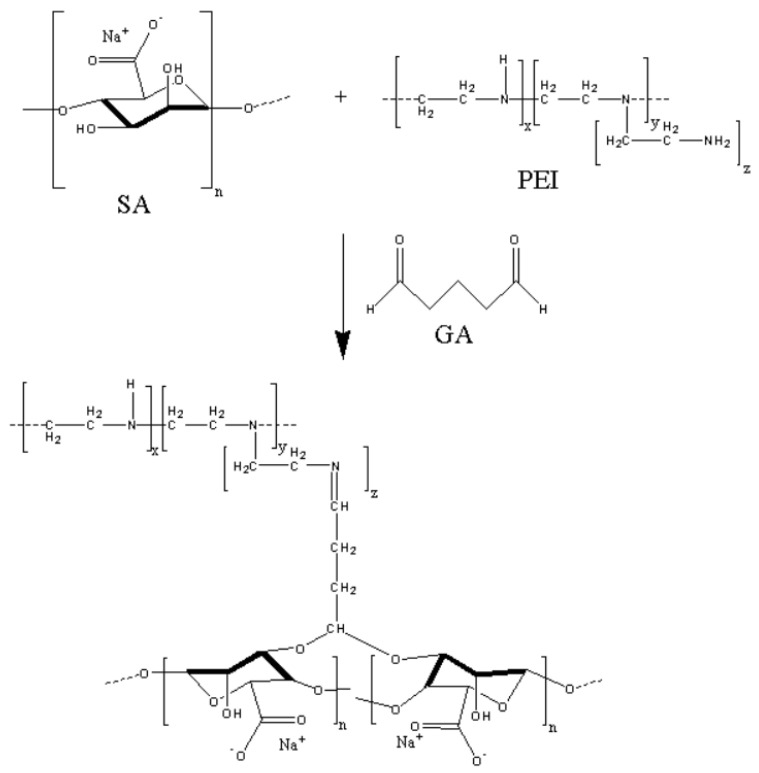
The scheme of covalent cross-linking between SA and PEI with GA.

The appearance of new peaks for supported membranes compared to dense membranes (Figure 9) may be due to the influence of the PAN substrate during FTIR analysis. The peak at 1166 cm^−1^ is attributed to the interaction of –OH of SA with the aldehyde group of GA forming acetal bonds [14]. It confirms the covalent cross-linking reaction, presented in Figure 12.

**Figure 13:**
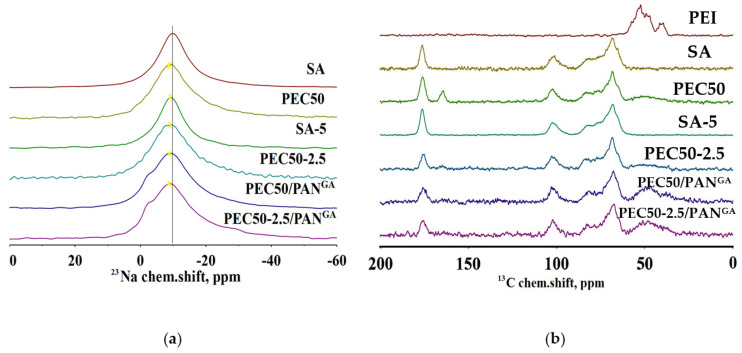
The NMR spectra for (**a**) ^23^Na and (**b**) ^13^C nuclei.

NMR spectra of ^23^Na and ^13^C nuclei of the membranes studied are shown in Figure 13.

The NMR spectra of ^23^Na nuclei for all membranes have one visible line around −9.5 ppm (Figure 13a), which corresponds to sodium ions of SA. It can be noted that the spectrum for the SA-5 membrane is noticeably narrowed and shifted. It may be associated with the formation of local homogeneity in the vicinity of sodium atoms, possibly due to crystallization in the region that contains GO, and the cross-linking of SA chains. For other membranes, this line shifts to the weak field region. Based on the results obtained by the computational experiment, such a shift may be explained by the formation of sodium coordination clusters, where the sodium atom acts as a coordination center between the amino group of PEI or the oxygen-containing groups of GO and the oxygen atoms of the ring, carboxylic, and hydroxyl groups of SA. Based on the topological analysis, their noncovalent nature was confirmed (the presence of bond critical points (3, −1) and bond paths). CPs and NCIplot surfaces for the characterization of interactions during the formation of model associates of SA with PEI are presented in Figure S2 of the Supplementary materials. Wiberg bond indexes (WBIs) calculated for this structure range from 0.18 to 0.36, which suggests a borderline nature between noncovalent interactions and dative bonding [60,61] (Tables S4 and S5 of the Supplementary Materials). This assumption can also be confirmed based on the NMR spectra of ^23^Na nuclei for GA cross-linked membranes (PEC50/PAN^GA^ and PEC50-2.5/PAN^GA^), where a low-intensity unresolved peak around −2 ppm is observed. The formation of additional peaks for GA cross-linked membranes may be explained by the inclusion of alcohol and water molecules into the inner sphere of the sodium ion, leading to the formation of new clusters and a signal in a weaker field. Also, this may be due to the fact that sodium ions were in the units that are directly involved in the SA and PEI with a GA bond.

The ^13^C NMR spectrum for the SA membrane (Figure 13b) corresponds to the spectrum of SA [48,62]. The spectrum of the SA-5 membrane with GO is practically no different from the spectrum of the SA membrane. The spectra of all PEC-based membranes show two additional broadened signals: (1) in the range of 30–60 ppm related to signals from PEI, and (2) around 164 ppm belonged to the carboxyl groups of SA involved in cross-linking with PEI. This also confirms the ionic cross-linking between SA and PEI in the PEC. Moreover, for the PEC50 membrane, the ratio of carboxyl groups involved and not involved in cross-linking, determined by the area under the peaks, is approximately 1:2.8. In the spectrum of the PEC50-2.5 membrane (based on the PEC modified with 2.5 wt.% GO), a signal around 164 ppm is observed and decreases; the ratio of carboxyl groups involved and not involved in ionic cross-linking is 1:7. Thus, it may be concluded that GO prevents and reduces close cross-linking of polymers (SA and PEI) during film synthesis. Earlier in the work [48], a similar effect was discovered, where the introduction of the carbon nanoparticle fullerenol into an SA matrix reduced the cross-linking effect of SA membranes and the number of bound polymer blocks in the membrane. In the spectra of membranes cross-linked with GA (PEC50/PAN^GA^ and PEC50-2.5/PAN^GA^), this line in the region of aliphatic carbons is more intense compared to the PEC50 membrane, which may be due to the presence of GA. For the PEC50-2.5/PAN^GA^ membrane, this line is also decreased compared to the PEC50/PAN^GA^ membrane due to GO modification (same as for non-cross-linked membranes). Moreover, for these PEC50/PAN^GA^ and PEC50-2.5/PAN^GA^ membranes, the ratios of carboxyl groups involved and not involved in cross-linking are 1:8 and 1:7, respectively. At the same time, the line of carboxyl carbons in the PEC50-2.5/PAN^GA^ membrane is slightly broadened, which may indicate a more amorphous structure of the film.

#### 3.2.2. Investigation of Membranes by Microscopic Methods

**Figure 14:**
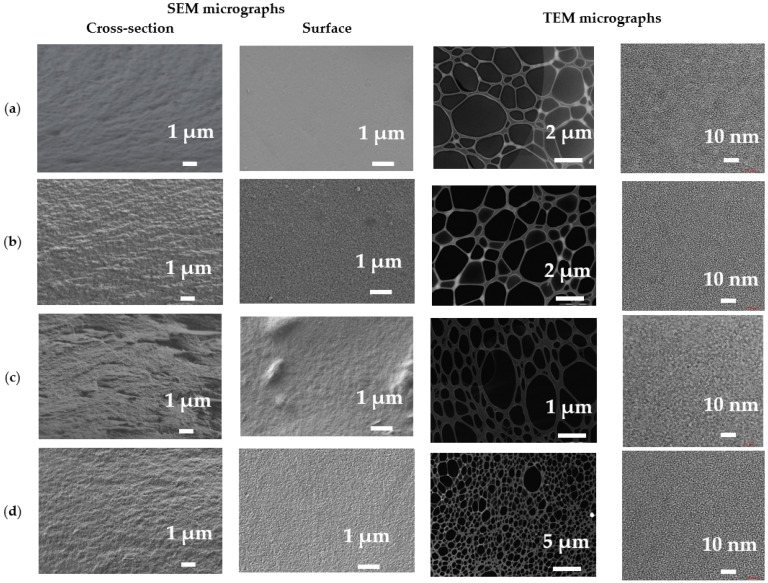
The cross-sectional and surface SEM micrographs and TEM images of (**a**) SA, (**b**) PEC50, (**c**) SA-5, and (**d**) PEC50-2.5 membranes.

The surface and cross-sectional structures of dense and supported membranes were studied by SEM, TEM, and AFM methods. The SEM micrographs of the cross-section, surface and TEM micrographs for dense membranes are presented in Figure 14.

The pristine SA membrane demonstrated a smooth structure of its cross-section and surface [48,63]. The formation of the PEC from SA and PEI led to a rougher cross-sectional and surface structure of the PEC50 membrane with “clusters” on the surface [14], which was explained by the ionic interaction between the anionic SA and cationic PEI. Also, these SEM images confirmed the uniform miscibility of polymers related to PEC formation [64]. The modification of SA with GO results in the significant formation of “plastic deformations” for the cross-section and surface, significantly increasing the roughness of the morphology. While for the PEC50-2.5 membrane, a slight increase in the structural roughness was observed compared to the PEC50 membrane. All these morphological changes may provide a permeability increase in the modified membranes during pervaporation.

TEM was used to study the detailed structure of the membrane matrix. This makes it possible to accurately determine the size distribution, shape, and dispersion state of nanoparticles [65]. TEM micrographs do not show the appearance of crystalline phases, agglomerates, pores, and clusters in the membrane matrix. All TEM micrographs were similar in structure. Thus, it may be concluded that the distribution of membrane components is uniform. 

**Figure 15:**
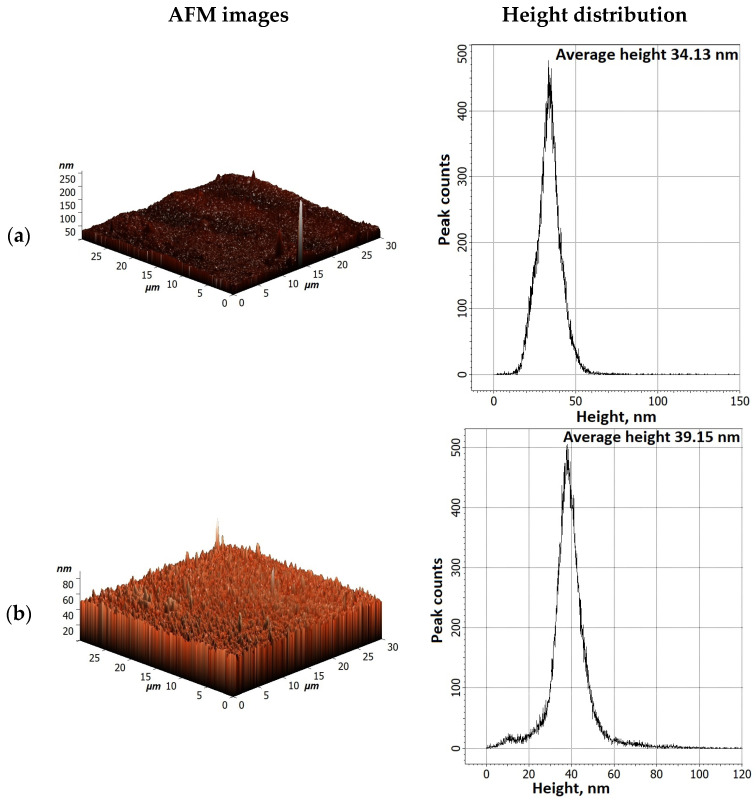

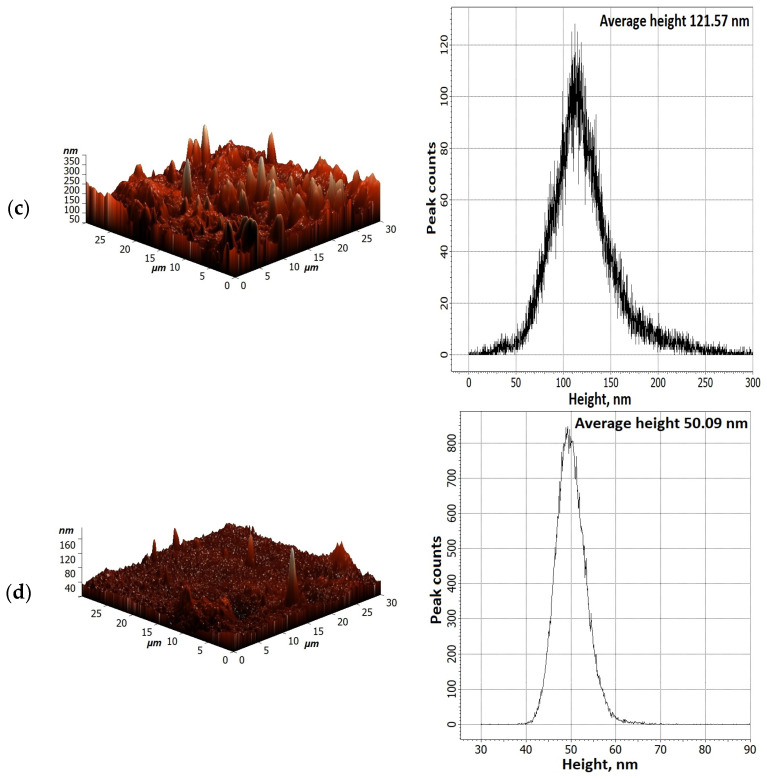
The surface AFM images and height distribution for (**a**) SA, (**b**) PEC50, (**c**) SA-5, and (**d**) PEC50-2.5 membranes.

The surface AFM images for dense membranes and height distribution are presented in Figure 15 to evaluate surface topography.

The presence of some data points with extreme heights in the AFM images of membranes was observed. To simplify the surface evaluation, the height distribution and average height were presented for membranes based on AFM images. It was demonstrated that the modification of SA with GO led to the highest average height of 121.57 nm. For PEC50 and PEC50-2.5 membranes, the average heights (39.15 and 50.09 nm, respectively) increased compared to the one of the SA membrane (34.13 nm). These changes would also affect the changes in surface roughness parameters, presented below.

**Figure 16:**
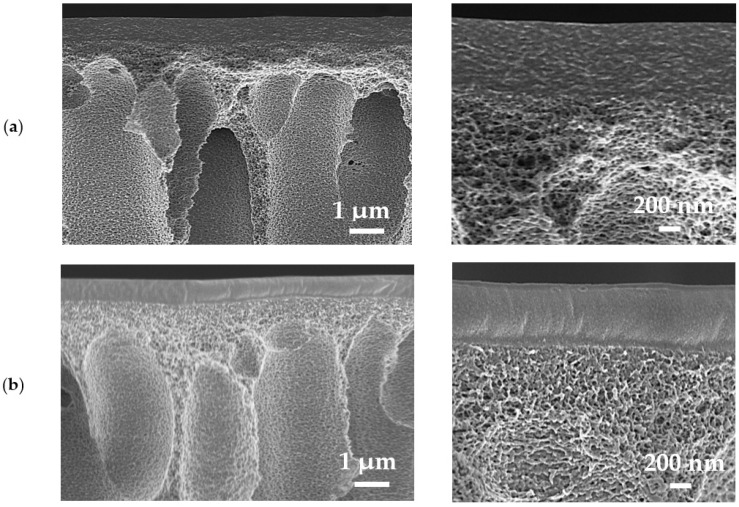
The cross-sectional SEM micrographs of supported (**a**) PEC50-2.5/PAN and (**b**) PEC50-2.5/PAN^GA^ membranes at different magnifications (20 and 50 KX).

The SEM micrographs of supported PEC50-2.5/PAN and cross-linked PEC50-2.5/PAN^GA^ membrane cross-sections are presented in Figure 16. The PEC50/PAN and PEC50/PAN^GA^ membrane micrographs were identical.

The uniform structure and good adhesion on the porous PAN substrate of the top thin dense layer based on the PEC/GO (2.5%) composite were observed. The thickness of the selective layers was found to be 500 ± 100 nm based on the obtained SEM data. The cross-linking with GA of supported PEC50/PAN membrane led to a smoother and more even structure of the cross-section and surface structure of the selective layer. This may also contribute to the reduced permeability of cross-linked membranes.

**Figure 17:**
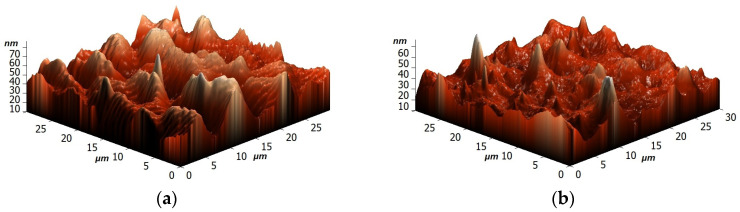

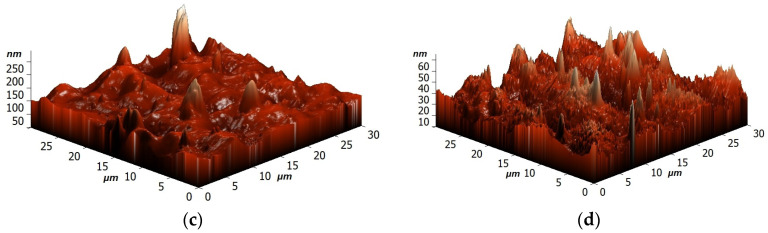
The surface AFM images of supported (**a**) PEC50/PAN, (**b**) PEC50/PAN^GA^, (**c**) PEC50-2.5/PAN, and (**d**) PEC50-2.5/PAN^GA^ membranes.

The AFM images of supported membranes (untreated PEC50/PAN and PEC50-2.5/PAN, and cross-linked PEC50/PAN^GA^ and PEC50-2.5/PAN^GA^) are presented in Figure 17. It is also worth noting that the surface SEM micrographs for supported membranes were not presented, since they were similar to the SEM micrographs of dense membranes.

#### 3.2.3. Investigation of Membrane Physicochemical Properties

The mass transport mechanism of pervaporation consists of three stages: (1) the sorption of components on the membrane, (2) diffusion through the membrane (the rate-determining stage), and (3) desorption from the opposite side of the membrane [66]. The first two stages are primarily responsible for the membrane permselectivity (component permeability coefficients ratio) [67,68]. Thus, the study of surface properties (in particular, roughness) is useful for the understanding of membrane performance [69]. It was found that the increase in surface roughness parameters of membranes led to the permeability rise due to an increase in the effective membrane area for contact with feed components [70], resulting to their faster sorption and penetration [71]. This effect was also confirmed for membranes from other polymers [71,72]. Surface topography of membranes were evaluated by the calculation of the average (R_a_) and root-mean-squared (R_q_) roughness based on the obtained AFM images (Table 2). Also, to assess the properties of the membrane surface, water contact angles were measured (Table 2).

The formation of the PEC from SA and PEI led to a slight increase in roughness characteristics of the PEC50 membrane due to the presence of “clusters” on the surface (Figure 14). The SA-5 and PEC50-2.5 membranes modified with GO have higher values of Ra and Rq compared to the unmodified membranes (SA and PEC50). This increase in surface roughness of the modified membranes provides a larger effective membrane surface for contact with components, leading to faster substance sorption and penetration and resulting in improved membrane permeability [31]. The roughness parameters of the supported PEC50/PAN and PEC50-2.5/PAN membranes were increased compared to dense membranes. It was due to the effect of the use of the PAN substrate for the creation of the thin dense selective layer. The application of cross-linking with GA for these supported membranes (PEC50/PAN^GA^ and PEC50-2.5/PAN^GA^) led to the decrease in surface roughness at the same level for both the unmodified and modified membranes. The obtained surface roughness parameters of membranes were in agreement with the SEM data.

The contact angles of water for the untreated membranes could not be measured due to the dissolution of the polymer matrix in water. For the PEC50/PAN^GA^ membrane, the contact angle of water was equal to 67°. This value is slightly lower compared to the previously developed SA membrane cross-linked with calcium chloride [63]. It may be due to addition of PEI with hydrophilic amine functional groups [73,74]. They form hydrogen bonds that are more favorable from an energetic point of view when interacting with water (according to Wiberg bond indexes, Table S4 of the Supplementary Materials). The introduction of GO into this membrane (cross-linked PEC50-2.5/PAN^GA^ membrane) led to a decrease in water contact angle of 64°, which was associated with the migration of GO-hydrophilic functional groups (oxygen-containing) to the membrane surface [31]. This surface hydrophilization by the GO addition and the increase in surface roughness collectively result in the increased performance of the modified membranes.

**Figure 18:**
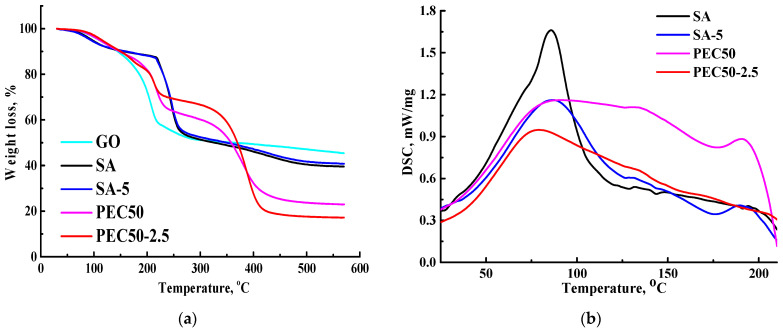
(**a**) TG curves for GO, SA, SA-5, PEC50, and PEC50-2.5 membranes, (**b**) DSC thermograms for SA, SA-5, PEC50, and PEC50-2.5 membranes.

The thermochemical properties of membranes were studied by TGA and DSC methods. The thermograms (TGs) for GO, SA, SA-5, PEC50, and PEC50-2.5 membranes, and DSC thermograms are presented in Figure 18.

There are three main steps for GO: <100 °C related to water evaporation (9% weight loss), 100–240 °C related to the decomposition of oxygen-containing groups (34% weight loss), and >240 °C related to carbon combustion [75]. All membranes started to lose residual water up to 100 °C, and there is an endothermic effect on the DSC thermograms related to this process [76]. For SA and SA-5 membranes, the TG curves were identical; only the modified SA-5 membrane had a slightly lower percentage of mass loss due to its more rigid structure (confirmed by SEM data, Figure 14). Three distinct stages were observed for them: <200 °C corresponds to absorbed water evaporation [77], 200–260 °C corresponds to carboxyl and hydroxyl groups decomposition, and >260 °C corresponds to the degradation of SA the backbone [78]. The endothermic effect occured at a slightly higher temperature for the SA-5 membrane (86.9 °C) compared to the SA membrane (85.8 °C), indicating a slight improvement in thermal stability [76]. The TG curves for PEC50 and PEC50-2.5 membranes have also three stages of weight loss and demonstrate higher thermal stability (less weight loss) in the range of 250–390 °C compared to SA-based membranes. This may be due to ionic cross-linking of the complex (SA/PEI), as well as the high decomposition temperature of PEI (about 300 °C) [79]. Comparing the endothermic effect of the SA and PEC50 membranes, it is broadening and blurring (without evidence maximum). This may be associated with kinetically inhibited water evaporation, which has also previously been observed in DSC thermograms of hydrophilic polymers such as PEI [80]. PEI in a PEC composition is capable of attracting significantly larger amounts of water. This effect may indicate the interaction between SA and PEI, which requires more energy for water evaporation [81], and this was also obtained for PEI-alginate nanocomposites in the work [82]. For the PEC50-2.5 membrane, the endothermic effect occurs at 79.3 °C. This may also indicate that the introduction of GO prevents and reduces the close cross-linking of the PEC. Also, it should be noted that the decrease in the endothermic peaks for membranes compared to the SA membrane may be associated with relatively decreased crystallinity due to ionic cross-linking and interactions, but is not comparable [14].

### 3.3. Performance Comparison with SA-Based Membranes

A comparison of the transport properties (in terms of the permeation flux and separation factor) of the developed supported PEC50-2.5/PAN and PEC50-2.5/PAN^GA^ membranes with the SA-based membranes described in the literature for the pervaporation dehydration of ethanol under conditions close to the present study is presented in Table 3.

It was shown that the comparison with other SA-based membranes was difficult due to other experimental conditions and the pervaporation temperature (25–77 °C). The developed membranes (PEC50-2.5/PAN and PEC50-2.5/PAN^GA^) were inferior in permeation flux to the SA-based membranes tested in the pervaporation dehydration of ethanol at higher temperatures. It is worth noting that the developed PEC50-2.5/PAN and PEC50-2.5/PAN^GA^ membranes demonstrated a high level of selectivity and high separation factor values.

## 4. Conclusions

Novel pervaporation membranes from SA were developed by the introduction of GO and PEI, forming a PEC with improved transport properties for the perspective-enhanced dehydration of bioalcohols, in particular, ethanol in a wide concentration range (4–90 wt.% water).

The introduction of GO (1–7 wt.%) into the SA matrix led to increased permeation flux, stability of up to 70 wt.% water in the feed, and high selectivity (99.99 wt.% water in the permeate) of membranes due to the cross-linking effect of GO, increased surface hydrophilization, and the roughness of modified membranes. The optimal concentration of GO was chosen as 5 wt.% with respect to the SA weight due to the highest permeation flux among all membranes.

The increase in PEI content (10–50%) in the PEC composition resulted in a rise of permeation flux and a slight reduction in selectivity of PEC-based membranes due to the expansion of the free volume between polymer chains, increased structure, and surface roughness. The PEC membrane from a 50/50 ratio of SA/PEI had optimal transport characteristics, and the highest permeation flux at a high level of selectivity.

The modification combination (the formation of the PEC and introduction of GO) led to more than 1.5 times increased permeation flux and selectivity for the PEC50-2.5 membrane compared to the pristine SA membrane. Supported membranes with a thin selective layer from this PEC/GO (2.5%) composite without and with cross-linking were developed. It allowed for an increase in the permeation flux of more than three times for the dehydration of ethanol with above 30 wt.% water in the feed, maintaining the same level of selectivity compared to a dense membrane. The cross-linked supported membrane PEC50-2.5/PAN^GA^ had improved transport properties in the pervaporation dehydration of ethanol (4–90 wt.% water), with more than two times increased permeation flux, higher selectivity (above 99.7 wt.% water in the permeate) and stability for separating diluted mixtures compared to the dense SA membrane. It will be promising for industrial applications for dehydration purposes even at elevated temperatures.

## Figures and Tables

**Table 1:** Membranes based on SA and PEC.

Membrane	Type	GO Content, wt.%	PEI Content, wt.%	Cross-Linking Method
SA	dense	0	-	-
SA-1	dense	1	-	-
SA-3	dense	3	-	-
SA-5	dense	5	-	-
SA-7	dense	7	-	-
PEC10	dense	0	10	-
PEC40	dense	0	40	-
PEC50	dense	0	50	-
PEC50-2.5	dense	2.5	50	-
PEC50/PAN	supported	0	50	-
PEC50-2.5/PAN	supported	2.5	50	-
PEC50/PAN^GA^	supported	0	50	1 wt.% GA and 0.5 wt.% H_2_SO_4_ for 5 min
PEC50-2.5/PAN^GA^	supported	2.5	50	1 wt.% GA and 0.5 wt.% H_2_SO_4_ for 5 min

**Table 2:** Surface parameters and water contact angle of SA and PEC-based membranes.

Membranes	Surface Roughness Parameters		Contact Angle of Water, °
	Ra, nm	Rq, nm	
SA	5.7	7.9	-
PEC50	6.4	10.8	-
SA-5	26.9	37.1	-
PEC50-2.5	7.6	11.9	-
PEC50/PAN	9.3	11.7	-
PEC50-2.5/PAN	14.5	21.5	-
PEC50/PAN^GA^	6.1	7.8	67 ± 3
PEC50-2.5/PAN^GA^	6.2	7.9	64 ± 3

**Table 3:** Comparison of transport properties of the pervaporation SA-based membranes for ethanol dehydration.

Membranes	Water Content in the Feed, wt.%	Temperature, °C	Permeation Flux, kg/(m^2^h)	Separation Factor (β)	Reference
PEC50-2.5/PAN	10	22	0.173	8991	This study
PEC50-2.5/PAN^GA^	10	22	0.136	8991	This study
SA—P4 *(40 ppm)/PVDF *	10	25	1.039	2638	[83]
HA */SA/PAN	10	30	~0.1	~400	[84]
SA/HA */PAN	10	30	~0.12	~100	
(SA-CS)_4 bilayers_/PAN	10	30	0.13	~4491	[85]
	10	60	0.210	1277	
SA—attapulgite nanorods (2%)/PAN	10	37	~0.2	~750	[86]
	10	76	1.356	2030	
(SA/PEI)_1.5 bilayers_/PAN	10pH = 3	60	1.203	1542	[22]
SA/PAA *-Fe_3_O_4_ (8%)/PAN	10	77	1.634	1044	[87]
SA—zwitterionic GO (2.5%)/PAN	10	77	2.140	1370	[88]

* PVDF—polyvinylidene fluoride, P4—amine-terminated polyamidoamine (PAMAM) dendrimers with 4 terminal primary amine groups, PAA—polyacrylic acid, HA—hyaluronic acid.

## Data Availability

Data are contained within the article and Supplementary Materials.

## Supplementary Materials

The following supporting information can be downloaded at: https://www.mdpi.com/article/10.3390/polym16091206/s1, Table S1: Cartesian atomic coordinates for all optimized equilibrium model structures; Table S2: The changes in thermodynamic potentials values during the association of components; Table S3: Calculated minimum values of enthalpies (∆H) and Gibbs free energies (ΔG) of reaction for hypothetical association processes; Figure S1: QTAIM distribution of bond critical points (CPs) (blue spheres) and bond paths for the associates (a) 1-H_2_O···SA, (b) 1-EtOH···SA, (c) 2-H_2_O···SA, and (d) 2-EtOH···SA; Figure S2: QTAIM distribution of bond CPs (blue spheres) and bond paths for the (a) PEI···SA and (b) 1-GO···EtOH associate; Table S4: The length of the interactions, WBI and FBO; Table S5: The interaction length, the ratio of interaction length to the sum of van der Waals radii and WBI values; Figure S3: Discriminating color bar of weak interaction; Figure S4: QTAIM distribution of bond CPs (blue spheres) and bond paths for the associates (a) 2-PEI···EtOH, (b) PEI···H_2_O, (c) GO···H_2_O; Table S6: Electron density ρ(r) (e/bohr^3^), Laplacian ∇^2^ρ(r) (e/bohr^5^), potential energy density V(r), Lagrangian kinetic energy G(r) (Hartree) at the bond CP (3, −1), corresponding to different noncovalent interactions. References [43,44,45,60,61,89,90,91,92,93,94,95,96] are cited in the supplementary materials.
