# Supplementary material for: Pervaporation Membranes Based on Polyelectrolyte Complex of Sodium Alginate/Polyethyleneimine Modified with Graphene Oxide for Ethanol Dehydration
Source: Polymers (Basel). 2024 Apr 25;16(9):1206. doi: 10.3390/polym16091206 (PMC11085317; doi:10.3390/polym16091206)
Supplement: Supplementary file 1 [file polymers-16-01206-s001.zip › polymers-2954334-supplementary.pdf]

Pervaporation Membranes Based on Polyelectrolyte Complex of Sodium Alginate/Polyethyleneimine Modified with Graphene Oxide for Ethanol Dehydration

Mariia Dmitrenko [1,*], Olga Mikhailovskaya [1], Roman Dubovenko [1], Anna Kuzminova [1], Danila Myznikov [1], Anton Mazur [1], Konstantin Semenov [2], Yury Rusalev [3], Alexander Soldatov [3], Sergey Ermakov [1] and Anastasia Penkova [1,*]

1 St. Petersburg State University, 7/9 Universitetskaya nab., St. Petersburg 199034, Russia; st113220@student.spbu.ru (O.M.); r.dubovenko@spbu.ru (R.D.); a.kuzminova@spbu.ru (A.K.); mailto:dan.myznikov@gmail.com (D.M.); a.mazur@spbu.ru (A.M.); s.ermakov@spbu.ru (S.E.)
2 Pavlov First Saint Petersburg State Medical University, L'va Tolstogo ulitsa 6–8, St. Petersburg 197022, Russia; semenov1986@yandex.ru
3 The Smart Materials Research Institute, Southern Federal University, 178/24 Sladkova St., Rostov-on-Don 344090, Russia; yuri.rusalev@gmail.com (Y.R.); soldatov@sfedu.ru (A.S.)
* Correspondence: m.dmitrienko@spbu.ru (M.D.); a.penkova@spbu.ru (A.P.); Tel.: +7-(812)-363-60-00 (ext. 3367) (M.D. & A.P.)

Theoretical Consideration

The structures of molecules were subjected to optimization using the B3LYP hybrid functional, known for its accuracy in computational chemistry [43-45]. The 6-311++G** basis set was employed for this purpose. The singlet state was determined to be the ground multiplicity state for all of the model systems under investigation. During the geometry optimization process, no symmetry restrictions were imposed. The initial procedure consisted of optimizing the molecular geometry of water, EtOH, PEI branched tetramer, and the individual unit of SA. Additionally, the geometries of paired associations were optimized. Also, the interactions between a preliminarily optimized GO fragment, containing epoxy and hydroxyl groups, and components of the feed were also considered. Associations exhibiting the most unfavorable Gibbs free energy were excluded from subsequent analysis. The Cartesian atomic coordinates can be found in Table S1.

Table S1. Cartesian atomic coordinates for all optimized equilibrium model structures.

Atom	x	y	z	Atom	x	y	z
H_2O				*2-EtOH···PEI*			
**O**	0.0000	0.0000	0.1171	**N**	5.6569	−2.3355	0.6418
**H**	0.0000	0.7635	−0.4683	**C**	4.3792	−1.6418	0.8490
**H**	0.0000	−0.7635	−0.4683	**C**	4.1928	−0.5660	−0.2228
EtOH				**N**	2.9154	0.1416	−0.1005
**C**	1.2163	−0.2414	−0.0216	**C**	2.9886	1.5254	−0.5776
**H**	1.2800	−0.9633	0.7997	**C**	1.8017	−0.6005	−0.6979
**H**	1.2820	−0.7903	−0.9642	**C**	3.5866	2.4812	0.4570
**H**	2.0829	0.4229	0.0544	**N**	3.6865	3.8306	−0.1130
**C**	−0.0796	0.5570	0.0469	**C**	0.4724	−0.3516	0.0179
**H**	−0.1305	1.1229	0.9871	**N**	−0.6080	−1.0739	−0.6547

**H**	−0.1269	1.2791	−0.7709
**O**	−1.2434	−0.2573	−0.1076
**H**	−1.2601	−0.9063	0.6030
SA			
**C**	−0.5027	−1.6910	−0.1024
**C**	0.6429	−1.3257	0.8425
**C**	1.7881	−0.6598	0.0465
**C**	1.2982	0.3452	−1.0564
**O**	−0.1095	0.5090	−1.1946
**C**	−1.1042	−0.4534	−0.7697
**O**	1.7656	1.6559	−0.7509
**O**	2.6495	0.0139	0.9638
**O**	0.2691	−0.5021	1.9371
**O**	0.0925	−2.5788	−1.0601
**C**	−2.1327	0.2476	0.1853
**O**	−3.2697	−0.1998	0.1717
**O**	−1.6423	1.1933	0.9067
**H**	−1.2988	−2.1970	0.4516
**H**	1.0344	−2.2469	1.2781
**H**	2.3728	−1.4307	−0.4529
**H**	1.6925	0.0552	−2.0336
**H**	−1.6516	−0.7615	−1.6636
**H**	2.4257	1.5210	−0.0416
**H**	2.1177	0.1270	1.7733
**H**	−0.4068	0.1672	1.6737
**H**	−0.6042	−2.9526	−1.6092
**Na**	−0.3328	2.5645	−0.1854
PEI			
**N**	−3.5198	−3.1306	−0.1812
**C**	−2.4760	−2.1701	−0.5616
**C**	−2.4806	−0.9875	0.4094
**N**	−1.4345	−0.0050	0.1136
**C**	−1.8188	1.3582	0.4905
**C**	−0.1288	−0.3927	0.6538
**C**	−2.7003	2.0376	−0.5597
**N**	−3.0868	3.3757	−0.0943
**C**	1.0379	0.1311	−0.1858
**N**	2.3138	−0.2333	0.4275
**C**	3.4820	0.1873	−0.3432
**C**	4.7770	−0.2650	0.3296
**N**	5.9382	0.1661	−0.4583
**H**	−3.6314	−3.8468	−0.8902
**H**	−3.2818	−3.6030	0.6854
**H**	−2.7028	−1.7941	−1.5628
**H**	−1.4694	−2.6115	−0.6085
**H**	−2.3968	−1.3602	1.4481
**C**	−1.8985	−0.9850	0.0260
**C**	−2.9383	−1.8445	−0.6911
**N**	−4.2407	−1.7743	0.0009
**H**	5.8679	−2.9514	1.4193
**H**	5.6287	−2.9101	−0.1950
**H**	4.4076	−1.1532	1.8262
**H**	3.5098	−2.3161	0.8564
**H**	4.3046	−1.0190	−1.2266
**H**	5.0106	0.1492	−0.1193
**H**	1.9821	1.8780	−0.8101
**H**	3.5650	1.5925	−1.5196
**H**	2.0132	−1.6699	−0.6416
**H**	1.6972	−0.3632	−1.7735
**H**	4.5473	2.0864	0.8205
**H**	2.9151	2.5205	1.3191
**H**	3.9317	4.5101	0.5987
**H**	4.4071	3.8723	−0.8269
**H**	0.2756	0.7303	0.0963
**H**	0.5544	−0.7284	1.0420
**H**	−0.7045	−0.7222	−1.6037
**H**	−1.7632	−1.3644	1.0462
**H**	−2.2775	0.0448	0.1162
**H**	−3.0866	−1.4631	−1.7074
**H**	−2.5514	−2.8677	−0.7798
**H**	−4.9325	−2.3256	−0.4977
**H**	−4.1658	−2.1819	0.9297
**C**	−7.3858	0.8089	0.7734
**H**	−7.3613	−0.2850	0.7956
**H**	−7.2558	1.1717	1.7961
**H**	−8.3758	1.1159	0.4194
**C**	−6.2903	1.3646	−0.1347
**H**	−6.4456	1.0096	−1.1652
**H**	−6.3509	2.4566	−0.1630
**O**	−4.9808	1.0532	0.3145
**H**	−4.8219	0.0931	0.2157
1-EtOH···SA			
**C**	−2.1277	1.4019	0.2539
**C**	−2.1901	0.1988	1.1974
**C**	−2.4956	−1.0908	0.3932
**C**	−1.7763	−1.1373	−0.9989
**O**	−0.8158	−0.1040	−1.2135
**C**	−0.9986	1.2550	−0.7737
**O**	−1.0092	−2.3205	−1.1089
**O**	−2.1122	−2.2189	1.1775
**O**	−1.0221	0.0125	1.9831
**O**	−3.4169	1.4327	−0.3754

**H**	−3.4571	−0.5048	0.3375	**C**	0.3663	1.7523	−0.1920
**H**	−0.9193	1.9649	0.6099	**O**	0.6315	2.9369	−0.3261
**H**	−2.3327	1.3715	1.4704	**O**	1.0530	0.8338	0.3977
**H**	−0.0607	−1.4819	0.6754	**H**	−1.9580	2.3187	0.8266
**H**	−0.0158	−0.0551	1.7019	**H**	−3.0091	0.3557	1.9017
**H**	−3.5577	1.3924	−0.8020	**H**	−3.5676	−1.1619	0.2134
**H**	−2.1183	2.1478	−1.4785	**H**	−2.5078	−1.1038	−1.8107
**H**	−3.5436	3.9001	−0.8322	**H**	−1.2258	1.8751	−1.6454
**H**	−3.7415	3.3172	0.6796	**H**	−1.2212	−2.8292	−0.3012
**H**	0.9382	1.2191	−0.3398	**H**	−1.4927	−1.8669	1.8434
**H**	0.9861	−0.3291	−1.1774	**H**	−0.2056	0.2575	1.4840
**H**	2.3650	0.1689	1.3598	**H**	−3.4945	2.2478	−0.8816
**H**	3.4124	−0.2758	−1.3354	**Na**	1.1761	−1.1445	−0.8717
**H**	3.5275	1.2768	−0.5059	**C**	5.2663	−0.1482	1.0586
**H**	4.8526	0.2075	1.3156	**H**	4.8473	0.6954	1.6144
**H**	4.7246	−1.3504	0.4966	**H**	5.0699	−1.0633	1.6228
**H**	6.8030	−0.0133	0.0398	**H**	6.3494	−0.0058	0.9965
**H**	5.9887	−0.3437	−1.3350	**C**	4.6651	−0.2332	−0.3372
GO				**H**	4.8845	0.6774	−0.9071
**C**	−4.6987	−2.0262	−0.5608	**H**	5.0870	−1.0772	−0.8879
**C**	−4.9276	−0.6938	−0.5049	**O**	3.2502	−0.4507	−0.3095
**C**	−3.3807	−2.5870	−0.3061	**H**	2.7830	0.3039	0.1116
**H**	−5.4950	−2.7063	−0.8428	***2-EtOH···SA***			
**C**	−3.8724	0.2232	−0.1612	**C**	2.0616	−1.5144	0.0842
**H**	−5.9022	−0.2876	−0.7545	**C**	0.8442	−1.4978	−0.8418
**C**	−3.1573	−4.0053	−0.5303	**C**	−0.4160	−1.2269	−0.0133
**C**	−2.3620	−1.7840	0.1191	**C**	−0.3048	0.0261	0.9167
**C**	−3.9733	1.5725	−0.3323	**O**	1.0087	0.4526	1.2377
**C**	−2.6058	−0.3558	0.3502	**C**	2.2477	−0.1709	0.7931
**C**	−2.8487	2.4658	−0.2188	**O**	−0.9184	1.1857	0.3465
**H**	−4.9196	1.9905	−0.6630	**O**	−1.5299	−1.1183	−0.8981
**O**	−2.1509	0.1455	1.6335	**O**	0.9303	−0.5847	−1.9260
**C**	−3.0175	3.8684	−0.5628	**O**	1.8113	−2.5843	1.0070
**C**	−1.6011	1.9845	0.0922	**C**	3.0761	0.7975	−0.1178
**C**	−1.9554	4.7001	−0.6979	**O**	4.2926	0.7002	−0.0291
**H**	−4.0204	4.2254	−0.7699	**O**	2.3819	1.5530	−0.8902
**C**	−1.4086	0.5799	0.4693	**H**	2.9680	−1.7156	−0.4949
**C**	−0.4399	2.9232	0.2593	**H**	0.7388	−2.4910	−1.2844
**C**	−0.6198	4.2489	−0.4528	**H**	−0.6019	−2.0893	0.6260
**H**	−2.0986	5.7221	−1.0335	**H**	−0.8112	−0.1875	1.8639
**C**	0.4941	4.9874	−0.7337	**H**	2.8313	−0.3274	1.7018
**C**	−1.9145	−4.5337	−0.4782	**H**	−1.8946	1.0755	0.3567
**H**	−4.0078	−4.6238	−0.7950	**H**	−1.1831	−0.6678	−1.6858
**C**	−0.7680	−3.7142	−0.1930	**H**	1.4015	0.2383	−1.6628
**H**	−1.7482	−5.5786	−0.7200	**H**	2.6094	−2.7348	1.5235
**C**	−0.9988	−2.3485	0.4505	**Na**	0.7797	2.5942	0.1085

**C**	0.1177	−1.3505	0.1525
**O**	−1.0328	−2.5919	1.8963
**C**	−0.0508	−0.0075	0.2764
**C**	1.0693	0.9227	0.1406
**C**	0.9066	2.2688	0.0094
**O**	−0.4154	3.3428	1.6824
**C**	2.0373	3.1436	−0.2516
**H**	−0.5731	2.5576	2.2247
**C**	1.8080	4.4607	−0.5818
**H**	0.3791	5.9938	−1.1244
**H**	2.6547	5.0983	−0.8132
**C**	0.5007	−4.1820	−0.3765
**C**	1.6598	−3.3494	−0.3154
**H**	0.6431	−5.2299	−0.6294
**C**	1.4282	−1.8853	−0.3070
**H**	−0.1870	−2.9843	2.1508
**C**	2.6263	−1.0084	−0.2809
**C**	2.4639	0.3567	0.3529
**C**	3.5994	1.2658	−0.0679
**O**	2.6019	0.0710	1.7744
**C**	3.3619	2.5845	−0.2853
**H**	4.1868	3.2468	−0.5335
**C**	2.9467	−3.8218	−0.3665
**C**	4.0910	−2.9709	−0.3075
**H**	3.1049	−4.8936	−0.4321
**O**	1.9303	−1.2096	−1.5128
**C**	3.9825	−1.6100	−0.2146
**C**	5.0863	−0.6940	−0.1727
**C**	4.9031	0.6561	−0.1632
**H**	2.6960	0.9133	2.2354
**H**	5.7626	1.3145	−0.2418
**H**	5.0761	−3.4257	−0.3243
**H**	6.0908	−1.1024	−0.2149
H_2O···EtOH			
**O**	2.3792	0.4770	−0.0269
**H**	3.0713	−0.0512	−0.4340
**H**	1.6178	−0.1210	0.0629
**C**	−1.4951	0.9467	0.1648
**H**	−1.8587	0.8333	1.1916
**H**	−0.6351	1.6199	0.1745
**H**	−2.2947	1.4120	−0.4195
**C**	−1.1185	−0.3987	−0.4380
**H**	−1.9804	−1.0761	−0.4599
**H**	−0.7662	−0.2773	−1.4636
**O**	−0.0280	−1.0331	0.2539
**H**	−0.2823	−1.1798	1.1713
**C**	−5.8352	−0.4035	0.1127
**H**	−5.5755	−1.2803	−0.4880
**H**	−5.8592	−0.7019	1.1634
**H**	−6.8395	−0.0827	−0.1799
**C**	−4.8363	0.7242	−0.1036
**H**	−4.8270	1.0353	−1.1560
**H**	−5.1057	1.5962	0.4961
**O**	−3.5131	0.3648	0.3120
**H**	−3.1594	−0.3616	−0.2324
EtOH···GO			
**C**	−4.8748	−1.7748	−0.7362
**C**	−5.0159	−0.4293	−0.7075
**C**	−3.5842	−2.4132	−0.5236
**H**	−5.7244	−2.4090	−0.9644
**C**	−3.8903	0.4276	−0.4383
**H**	−5.9735	0.0319	−0.9256
**C**	−3.4576	−3.8464	−0.7204
**C**	−2.4979	−1.6663	−0.1683
**C**	−3.9088	1.7750	−0.6478
**C**	−2.6384	−0.2198	0.0280
**C**	−2.2467	−4.4491	−0.7052
**H**	−4.3534	−4.4176	−0.9375
**C**	−1.1598	−2.3038	0.1195
**C**	−2.7272	2.6016	−0.5927
**H**	−4.8394	2.2444	−0.9541
**O**	−2.1002	0.2785	1.2950
**C**	−2.8172	4.0099	−0.9405
**C**	−1.5044	2.0467	−0.3153
**C**	−1.3929	0.6370	0.0670
**C**	−1.7067	4.7726	−1.1025
**H**	−3.8000	4.4299	−1.1241
**C**	−0.2790	2.9087	−0.1739
**C**	−0.3959	4.2407	−0.8868
**H**	−1.7942	5.8015	−1.4370
**C**	−0.0824	−0.0355	−0.1620
**C**	1.0149	2.1706	−0.4632
**O**	−0.1925	3.3057	1.2400
**C**	0.7560	4.9046	−1.2014
**C**	2.0383	4.2980	−1.0775
**H**	0.6952	5.9140	−1.5968
**C**	−1.0410	−3.6993	−0.4821
**H**	−2.1549	−5.5068	−0.9308
**C**	0.1930	−4.2500	−0.6865
**C**	0.0005	−1.3887	−0.2531
**O**	−1.1210	−2.4891	1.5860
**C**	1.2679	−2.0169	−0.7095

H_2O···PEI			
**N**	−3.2444	−2.7585	−0.1178
**C**	−2.1188	−1.8787	−0.4979
**C**	−2.0183	−0.7039	0.4776
**N**	−0.9079	0.1929	0.1532
**C**	−1.1922	1.5878	0.5066
**C**	0.3736	−0.2779	0.6837
**C**	−2.0593	2.2970	−0.5360
**N**	−2.3575	3.6634	−0.0846
**C**	1.5646	0.1470	−0.1772
**N**	2.8176	−0.3057	0.4250
**C**	4.0040	0.0178	−0.3650
**C**	5.2703	−0.5219	0.2979
**N**	6.4501	−0.1875	−0.5088
**H**	−3.3451	−3.5139	−0.7891
**H**	−3.0663	−3.1932	0.7840
**H**	−2.3198	−1.4845	−1.4967
**H**	−1.1632	−2.4172	−0.5456
**H**	−1.9350	−1.0867	1.5130
**H**	−2.9559	−0.1484	0.4305
**H**	−0.2515	2.1355	0.5871
**H**	−1.6763	1.6559	1.4989
**H**	0.3648	−1.3693	0.7225
**H**	0.5241	0.0666	1.7248
**H**	−2.9583	1.7007	−0.7453
**H**	−1.4931	2.3594	−1.4693
**H**	−2.7894	4.2011	−0.8282
**H**	−3.0157	3.6503	0.6887
**H**	1.5448	1.2374	−0.3433
**H**	1.4654	−0.3193	−1.1624
**H**	2.9109	0.1030	1.3512
**H**	3.8873	−0.4499	−1.3505
**H**	4.1281	1.0989	−0.5415
**H**	5.3932	−0.0452	1.2771
**H**	5.1395	−1.5985	0.4789
**H**	7.3056	−0.4230	−0.0182
**H**	6.4525	−0.7104	−1.3792
**O**	−5.4160	−0.8575	−0.2084
**H**	−6.2792	−1.2166	0.0138
**H**	−4.7875	−1.6080	−0.1410
1-H_2O···SA			
**C**	−1.3097	1.5494	0.0693
**C**	−1.6490	0.3979	1.0181
**C**	−1.9911	−0.8767	0.2046
**C**	−1.0947	−1.0576	−1.0685
**O**	0.0049	−0.1526	−1.1620
**H**	−0.4308	−3.1393	1.7728
**C**	1.0927	0.8190	−0.3226
**C**	2.4423	0.1707	−0.0878
**C**	2.1944	2.9704	−0.7442
**H**	−0.0454	2.5043	1.7769
**C**	3.4781	2.3239	−0.7883
**H**	2.9171	4.8819	−1.3301
**C**	1.4033	−3.4918	−0.6742
**H**	0.2621	−5.3116	−0.9121
**C**	2.6584	−4.0461	−0.7220
**C**	2.5158	−1.2140	−0.7043
**O**	1.8062	−1.4080	−1.9337
**C**	3.8303	−1.9004	−0.6255
**C**	3.6290	0.9993	−0.5381
**O**	2.6083	−0.0427	1.3402
**C**	4.8883	0.3060	−0.6218
**H**	1.8122	0.2607	1.8142
**H**	4.3423	2.9254	−1.0566
**C**	3.8536	−3.2683	−0.6894
**H**	2.7486	−5.1273	−0.7574
**H**	4.8082	−3.7844	−0.6968
**C**	4.9879	−1.0540	−0.5986
**H**	5.9656	−1.5241	−0.6278
**H**	5.7886	0.9055	−0.7153
**C**	0.6245	−0.3997	4.9444
**H**	−0.1663	−1.1310	4.7540
**H**	1.5698	−0.8104	4.5823
**H**	0.7011	−0.2582	6.0269
**C**	0.3179	0.9271	4.2647
**H**	−0.6184	1.3534	4.6422
**H**	1.1125	1.6532	4.4490
**O**	0.2390	0.8047	2.8321
**H**	−0.5375	0.2684	2.5966
1-PEI···SA			
**C**	6.1891	1.3185	0.2366
**C**	6.7043	−0.0205	−0.2928
**C**	6.4407	−1.1381	0.7403
**C**	5.0423	−1.0684	1.4513
**O**	4.2690	0.1095	1.2757
**C**	4.6920	1.2860	0.5427
**O**	4.2171	−2.1427	1.0040
**O**	6.5751	−2.3986	0.0847
**O**	6.1920	−0.3889	−1.5615
**O**	6.9642	1.5620	1.4182
**C**	3.8217	1.4499	−0.7436
**O**	3.4062	2.5761	−0.9974

**C**	−0.0729	1.2339	−0.7814	**O**	3.6093	0.3627	−1.3948
**O**	−0.4684	−2.3255	−1.0442	**H**	6.3662	2.1005	−0.5094
**O**	−1.8596	−2.0119	1.0580	**H**	7.7863	0.0623	−0.4160
**O**	−0.6349	0.1071	1.9685	**H**	7.1992	−1.0824	1.5202
**O**	−2.4837	1.7057	−0.7405	**H**	5.1755	−1.1774	2.5309
**C**	1.2471	1.5895	−0.0210	**H**	4.4503	2.1306	1.1917
**O**	1.6711	2.7282	−0.1385	**H**	4.8331	−2.7640	0.5696
**O**	1.7285	0.6178	0.6785	**H**	6.5031	−2.1935	−0.8663
**H**	−1.1136	2.4613	0.6415	**H**	5.2202	−0.2026	−1.6090
**H**	−2.5342	0.6753	1.5932	**H**	6.7267	2.4258	1.7717
**H**	−3.0280	−0.8296	−0.1259	**Na**	2.4750	−1.0033	−0.0036
**H**	−1.6952	−0.9679	−1.9777	**N**	−8.2331	3.2421	1.0632
**H**	−0.0989	1.8432	−1.6891	**C**	−7.2837	2.1237	1.1133
**H**	−0.8518	−2.7765	−0.2658	**C**	−7.6466	1.0844	0.0506
**H**	−1.3035	−1.7101	1.8001	**N**	−6.7084	−0.0425	0.0182
**H**	0.2673	0.2349	1.5882	**C**	−7.3413	−1.2928	−0.4094
**H**	−2.3922	2.5059	−1.2677	**C**	−5.4982	0.2627	−0.7504
**Na**	1.7862	−1.4113	−0.5223	**C**	−8.1373	−1.9745	0.7053
**O**	3.8147	−0.9197	0.3531	**N**	−8.8116	−3.1656	0.1730
**H**	3.3807	−0.0949	0.6729	**C**	−4.2377	−0.4000	−0.1843
**H**	4.7631	−0.8279	0.4646	**N**	−3.0914	−0.0815	−1.0350
2-H₂O···SA				**C**	−1.7752	−0.2349	−0.4271
**C**	0.5512	1.7305	−0.0638	**C**	−0.6958	0.2720	−1.3842
**C**	−0.8286	1.1537	0.2815	**N**	0.6163	0.4020	−0.7168
**C**	−1.3875	0.4640	−0.9717	**H**	−8.1062	3.8640	1.8538
**C**	−0.4178	−0.5919	−1.5732	**H**	−8.1000	3.7963	0.2230
**O**	0.9612	−0.4240	−1.2975	**H**	−7.3609	1.6510	2.0959
**C**	1.5357	0.6235	−0.4620	**H**	−6.2345	2.4318	0.9909
**O**	−0.7703	−1.8938	−1.0918	**H**	−7.7167	1.5749	−0.9384
**O**	−2.6340	−0.1945	−0.7749	**H**	−8.6479	0.7144	0.2774
**O**	−0.7949	0.3286	1.4400	**H**	−6.5666	−1.9887	−0.7388
**O**	0.3291	2.6719	−1.1198	**H**	−7.9995	−1.1275	−1.2840
**C**	2.2095	0.0223	0.8162	**H**	−5.3278	1.3410	−0.7395
**O**	3.2214	0.5855	1.2075	**H**	−5.6194	−0.0156	−1.8131
**O**	1.5915	−0.9779	1.3395	**H**	−8.8248	−1.2524	1.1692
**H**	0.9595	2.2366	0.8166	**H**	−7.4404	−2.2932	1.4852
**H**	−1.4912	1.9892	0.5214	**H**	−9.2352	−3.7076	0.9178
**H**	−1.5154	1.2470	−1.7221	**H**	−9.5572	−2.9040	−0.4656
**H**	−0.5090	−0.6069	−2.6631	**H**	−4.3938	−1.4831	−0.0531
**H**	2.3332	1.0614	−1.0642	**H**	−4.0528	0.0092	0.8132
**H**	−1.7120	−1.8115	−0.8422	**H**	−3.1429	−0.6064	−1.9028
**H**	−3.0005	−0.0364	0.1189	**H**	−1.7579	0.3777	0.4824
**H**	0.0145	−0.2401	1.4689	**H**	−1.5439	−1.2694	−0.1158
**H**	1.1435	3.1629	−1.2691	**H**	−0.5807	−0.4263	−2.2198
**Na**	1.1157	−2.5414	−0.0736	**H**	−1.0381	1.2249	−1.8036
**O**	−3.4148	−0.0938	1.8875	**H**	1.3009	0.7494	−1.3920
**H**	−3.9158	0.4946	2.4592	**H**	0.5485	1.1466	−0.0250

**H**	−2.4684	0.0293	2.0990	***2-PEI···SA***			
H_2O···GO				**C**	3.3509	−1.4954	−1.6980
**C**	1.9277	4.7682	−0.6165	**C**	3.8931	−2.1185	−0.4108
**C**	3.0423	4.0088	−0.5068	**C**	5.0932	−1.3009	0.1127
**C**	0.5984	4.2121	−0.4165	**C**	4.9201	0.2568	0.0268
**H**	2.0046	5.8116	−0.9026	**O**	3.8478	0.7608	−0.7563
**C**	2.9529	2.6148	−0.1585	**C**	2.9011	−0.0479	−1.4974
**H**	4.0215	4.4258	−0.7176	**O**	4.7413	0.7973	1.3349
**C**	−0.5664	5.0376	−0.6930	**O**	5.3380	−1.6741	1.4684
**C**	0.4484	2.9261	0.0140	**O**	2.9278	−2.2918	0.6126
**C**	3.9986	1.7478	−0.2830	**O**	4.4374	−1.5872	−2.6287
**C**	1.6344	2.1106	0.3003	**C**	1.4874	0.0389	−0.8412
**C**	−1.8121	4.5140	−0.6847	**O**	0.5264	0.1565	−1.6019
**H**	−0.4076	6.0773	−0.9572	**O**	1.4783	−0.0077	0.4380
**C**	−0.9197	2.3441	0.3076	**H**	2.4899	−2.0735	−2.0500
**C**	3.8519	0.3190	−0.1728	**H**	4.2586	−3.1189	−0.6520
**H**	4.9700	2.1352	−0.5757	**H**	5.9756	−1.5583	−0.4717
**O**	1.6278	1.4364	1.5862	**H**	5.8246	0.7025	−0.3960
**C**	4.9899	−0.5375	−0.4668	**H**	2.8229	0.4316	−2.4758
**C**	2.6261	−0.2414	0.0884	**H**	5.0329	0.0881	1.9397
**C**	1.4695	0.5990	0.4165	**H**	4.5163	−2.1101	1.7605
**C**	4.8544	−1.8798	−0.6005	**H**	2.3578	−1.4881	0.6888
**H**	5.9515	−0.0663	−0.6376	**H**	4.1369	−1.2761	−3.4889
**C**	2.4861	−1.7265	0.2581	**Na**	2.6472	1.7422	1.1891
**C**	3.5919	−2.5243	−0.4032	**N**	−7.7927	0.5808	−1.9288
**H**	5.7011	−2.4895	−0.8987	**C**	−6.7398	0.7421	−0.9167
**C**	0.1111	0.0328	0.1715	**C**	−5.8445	−0.4982	−0.9000
**C**	1.0903	−2.2411	−0.0442	**N**	−4.7377	−0.3910	0.0538
**O**	2.7047	−2.0290	1.6974	**C**	−4.3413	−1.6932	0.5983
**C**	3.3591	−3.8399	−0.6860	**C**	−3.6039	0.3636	−0.4935
**C**	2.0611	−4.4159	−0.5829	**C**	−5.2394	−2.1423	1.7528
**H**	4.1771	−4.4602	−1.0393	**N**	−4.8249	−3.4705	2.2234
**C**	−2.0324	3.1214	−0.4001	**C**	−2.7768	1.0598	0.5879
**H**	−2.6685	5.1218	−0.9583	**N**	−1.6924	1.8265	−0.0235
**C**	−3.2391	2.5370	−0.6453	**C**	−0.8166	2.4587	0.9503
**C**	−0.9648	0.8469	0.0036	**C**	0.1453	3.4175	0.2461
**O**	−1.1386	2.5556	1.7259	**N**	1.2975	3.7663	1.1273
**C**	−2.2403	0.2751	−0.5095	**H**	−8.4674	1.3359	−1.8764
**H**	−1.7171	1.8516	2.0805	**H**	−7.3989	0.5974	−2.8650
**C**	0.0068	−1.4199	0.0373	**H**	−7.2160	0.8380	0.0628
**C**	−1.3755	−2.0291	0.1944	**H**	−6.1263	1.6434	−1.0621
**C**	0.9427	−3.6632	−0.3019	**H**	−5.4717	−0.6982	−1.9228
**H**	2.3336	−1.2978	2.2099	**H**	−6.4692	−1.3527	−0.6320
**C**	−0.3777	−4.2263	−0.3848	**H**	−3.3190	−1.6308	0.9739
**H**	1.9393	−5.4692	−0.8132	**H**	−4.3329	−2.4703	−0.1892
**C**	−3.4513	1.1271	−0.5849	**H**	−3.9865	1.1356	−1.1643

**H**	−4.0773	3.1621	−0.9430	**H**	−2.9479	−0.2838	−1.1021
**C**	−4.6815	0.5306	−0.6888	**H**	−6.2946	−2.0963	1.4436
**C**	−2.4472	−1.1974	−0.4773	**H**	−5.1208	−1.4336	2.5768
**O**	−2.0480	−0.5690	−1.6968	**H**	−5.3396	−3.7391	3.0550
**C**	−3.8236	−1.7521	−0.4670	**H**	−5.0061	−4.1783	1.5182
**C**	−1.4994	−3.4798	−0.2173	**H**	−2.4026	0.3183	1.3141
**O**	−1.7217	−1.9420	1.6236	**H**	−3.4300	1.7440	1.1407
**C**	−2.8411	−3.9912	−0.3595	**H**	−1.1262	1.2058	−0.6051
**H**	−1.2056	−2.6127	2.0876	**H**	−1.4323	3.0207	1.6645
**H**	−0.4661	−5.2806	−0.6322	**H**	−0.2360	1.7213	1.5334
**C**	−4.8689	−0.8823	−0.6189	**H**	0.5405	2.9316	−0.6497
**H**	−5.5573	1.1615	−0.7991	**H**	−0.4037	4.3054	−0.0848
**H**	−5.8794	−1.2737	−0.6770	**H**	1.8083	4.5450	0.7170
**C**	−3.9355	−3.1818	−0.4184	**H**	0.9448	4.1189	2.0148
**H**	−4.9257	−3.6190	−0.4978				
**H**	−2.9639	−5.0670	−0.4348				
**O**	−2.6810	0.4508	2.8567				
**H**	−2.6137	0.4171	3.8155				
**H**	−2.3629	−0.4104	2.5308				

The investigation of potential interactions between the constituents of the composite material and the molecules in the mixture being separated enables the identification of potential molecular interaction mechanisms and provides insights into the observed experimental outcomes. Thus, potential interactions between the monomeric units of SA, PEI tetramer, water, and EtOH were investigated. Additionally, the interaction between GO and components of the feed was also considered.

In order to evaluate the thermodynamic stability of various hypothetical supramolecular associations, quantum chemical calculations were conducted. In order to validate the optimized model structures, Hessian matrices were computed and analyzed. The absence of any imaginary frequencies confirmed that these structures represent true minima on the potential energy surface. Thermodynamic parameters were evaluated at standard conditions of 298.15 K and 1.00 atm. By comparing the thermodynamic properties of the associations and individual components, the differences in enthalpies and Gibbs free energies were determined. The obtained results, presented in Table S2, offer valuable insights into the most favorable interactions observed among the associates.

Table S2. The changes of thermodynamic potentials values during the association of components.

B3LYP/6-311++G**			ΔH(kJ/mol)	ΔG(kJ/mol)	$ΔH_{max}$(kJ/mol)	$ΔG_{mostnegative}$ (kJ/mol)
H_2O	EtOH	1*	−18.7	10.7	−18.7	10.7
	PEI	1	−25.6	7.5	−25.6	7.5
	SA	1	−72.0	−34.3	−72.0	−34.3
		2	−33.0	5.6		
	GO	1	−35.4	5.0	−35.4	5.0
EtOH	PEI	1	−12.8	28.1	−22.0	12.5
		2	−20.9	12.5		
		3	−22.0	13.8		
	SA	1	−72.1	−32.4	−72.1	−32.4
		2	−29.5	10.2		
	GO	1	−44.8	3.0	−44.8	3.0
		2	−35.4	7.2		
PEI	SA	1	−56.2	−11.9	−70.6	−25.3
		2	−70.6	−25.3		
		3	−26.8	29.6		
		4	−23.7	24.1		
		5	−6.8	38.6		

** the 3rd column (with numbers 1, 2, 3, 4) shows the type of hypothetical supramolecular association process.*

These interactions, characterized by the lowest values of potentials, are summarized in Table S3.

Table S3. Calculated minimum values of enthalpies (ΔH) and Gibbs free energies (ΔG) of reaction for hypothetical association processes.

ΔH, (kJ/mol)					
B3LYP/6-311++G**	H_2O	EtOH	PEI	SA	GO
H_2O	~	−18.7	−25.6	−72.0	−35.4
EtOH		~	−22	−72.1	−44.8
PEI			~	−70.6	-
SA				~	-
**ΔG, (kJ/mol)**					
B3LYP/6-311++G**	H_2O	EtOH	PEI	SA	GO
H_2O	~	10.7	7.5	−34.3	5.0

**EtOH**		~	12.5	−32.4	3.0
**PEI**			~	−25.3	-
**SA**				~	-

Based on the results presented in Table S3, it can be concluded that SA exhibits the highest affinity towards the components of the mixture being separated. As a result of the optimization procedure using the Bader quantum theory of atoms-in-molecules (QTAIM) method, it was shown that the primary mechanism responsible for this energetic advantage (−34.3 and −32.4 for water and EtOH, respectively) was due to the formation of coordination clusters with sodium (Figure S1 a, b), as confirmed by the values of the Wiberg bond indexes (WBI), presented below in Table S4.

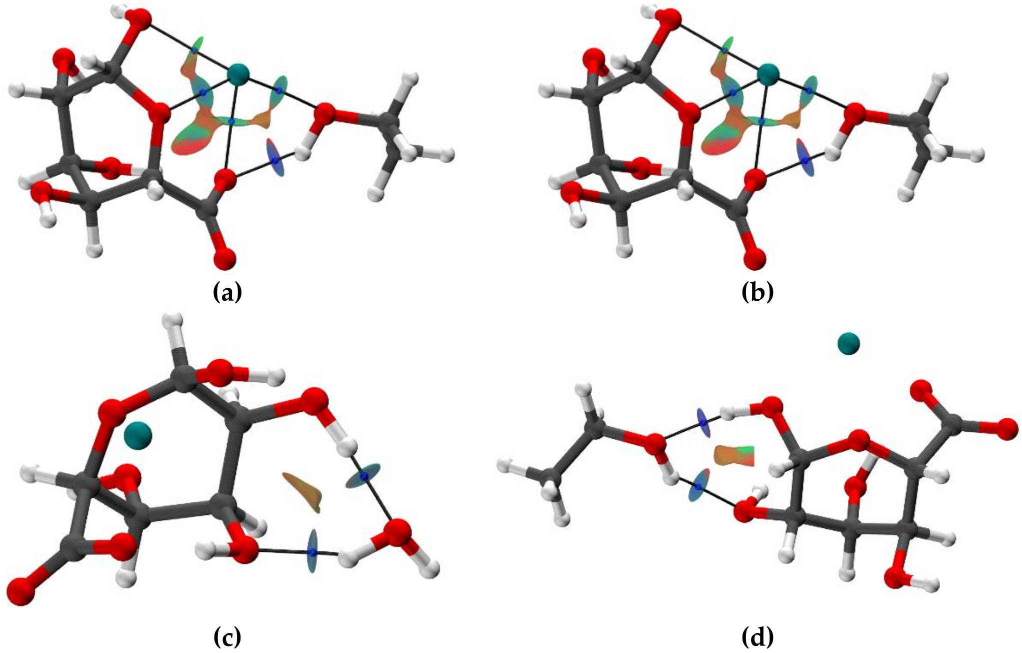

Figure S1. QTAIM distribution of bond critical points (CPs) (blue spheres) and bond paths for the associates **(a)** 1-H_2O···SA, **(b)** 1-EtOH···SA, **(c)** 2-H_2O···SA, and **(d)** 2-EtOH···SA. The NCIplot index isosurfaces are also represented using an isovalue of 0.5 ($e^{1/3}$ bohr)$^{-1}$, the color range is −0.04 e/bohr^3 ≤ sign($\lambda 2$)ϱ ≤ 0.02 e/bohr^3. CPs and NCIplot surfaces for characterization of intermolecular interactions are presented for clarity.

Analyzing classical hydrogen bonding interactions, the changes in isobaric-isothermal potential for association between water and EtOH with the hydroxyl groups of SA, as depicted in Figure S1 c, d, were determined to be 5.6 and 10.2 kJ/mol, respectively (Table S2). Consequently, it can be deduced that GO interactions (5 and 3 kJ/mol for association between water and EtOH, respectively) are more favorable.

The employed computational data allows proposing a modified version of the SA-PEI cross-linking. In this hypothesis, the Na^+ ion is directly involved in the cross-linking

process, acting as a coordination center between the amino group of PEI and the oxygen atoms of the ring, carboxylic, and hydroxyl groups of SA. A similar "pseudo bridged" unidentate arrangement for the carboxylate-metal complex, wherein one carboxylate oxygen interacts with the metal ion and the other participates in a hydrogen bond with an adjacent hydroxyl group, was proposed in the study [89]. In the presented system, this carboxylate-metal complex is formed utilizing the NH group of the PEI polymer chain (Figure S2 a). This notion is further supported by the slight shift towards a weaker field observed in the NMR spectra of ^{23}Na nuclei of the PEC50 membrane (Figure 13).

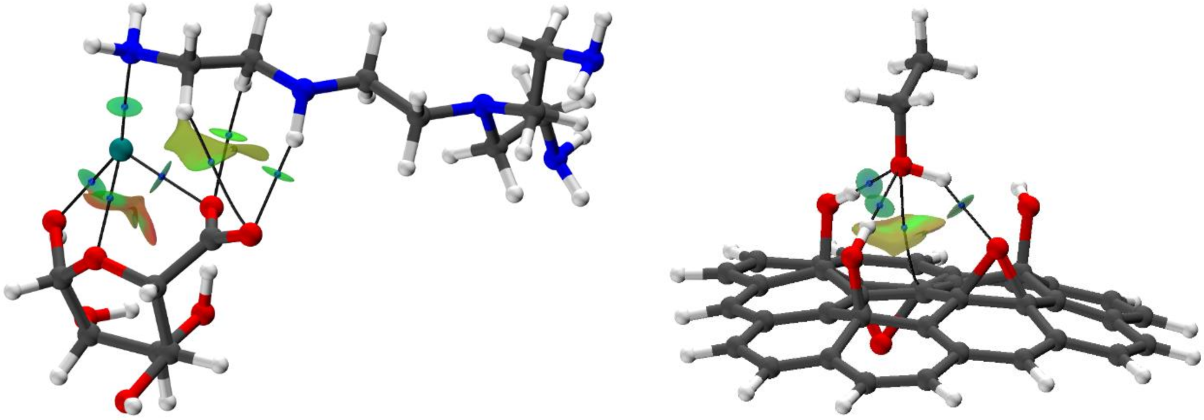

(a) **(b)**

Figure S2. QTAIM distribution of bond CPs (blue spheres) and bond paths for the **(a)** PEI···SA and **(b)** 1-GO···EtOH associate. The NCIplot index isosurfaces are also represented using an isovalue of 0.5 $(e^{1/3}\ bohr)^{-1}$, the color range is -0.04 e/bohr^3 $\leq$ sign($\lambda 2$)ϱ $\leq$ 0.02 e/bohr^3. CPs and NCIplot surfaces for characterization of intermolecular interactions are presented for clarity.

Based on the SA···PEI, SA···H2O, and SA···EtOH systems, where a coordination cluster with sodium is formed (Figures S1 a, b and S2 a), it is plausible to assume a similar interaction between GO containing hydroxyl groups and SA, causing cross-linking of polymer chains (shift in the NMR spectra of ^{23}Na nuclei, Figure 13).

Moreover, a noteworthy consideration is the examination of the association between GO and the components of the feed. The interaction between GO and EtOH is more favorable compared to water, which can be attributed not only to hydrogen bonding but also to the noncovalent interaction between the oxygen of the alcohol group and the carbon framework of graphene (as confirmed by the topological analysis, which revealed a bond critical point (3, −1), Figure S2 b).

Despite this fact, an increase in both membrane performance and selectivity was observed for the GO-modified membranes (Figure 4), likely attributed to an increase in the aforementioned membrane surface roughness and hydrophilization (confirmed by AFM and contact angle data, Table 2). GO in the membrane preferentially interacts with ethanol, preventing its further penetration. Also, due to the fact that water penetrates more easily through the GO-modified membrane due to its smaller size compared to EtOH, the water content in the permeate turns out to be higher.

A topological analysis was carried out, the lengths and orders of bonds were determined using a multifunctional wavefunction analyzer (Multiwfn) in order to evaluate the strength of interactions between components of composites and feed. The WBI [90, 91] and Fuzzy bond order (FBO) [92] were utilized to assess the strength of the bonding. These indexes were chosen due to their resistance to changes in the basis set and effective quantification of bonding strength. The results of post-processing of wave functions are presented in Table S4.

Table S4. The length of the interactions, WBI and FBO.

B3LYP/6-311++G**					
**Associate**		**Interaction**	**d, Å**	**WBI**	**FBO**
**PEI**	**H_2O**	$N(NH_2)$///H	1.9250	0.146	0.107
		$H(CH_2)$///O	2.6388	0.018	0.024
	**EtOH**	$N(NH_2)$///H(OH)	1.9675	0.130	0.098
	**SA**	H(NH)///$O(CO_2)$	2.1968	0.051	0.047
		$H(CH_2)$///$O(CO_2)$	2.9340	0.009	0.013
		$H(CH_2)$///$O(CO_2)$	2.6698	0.016	0.020
		$N(NH_2)$///Na	2.4336	0.340	0.490
**SA**	**1-H_2O**	$O(CO_2)$///H	1.7994	0.124	0.084
		Na///O	2.2633	0.344	0.489
	**2-H_2O**	H(OH)///O(OH)	1.8233	0.127	0.096
		O(OH)///H(OH)	1.8173	0.117	0.082
	**1-EtOH**	$O(CO_2)$///H(OH)	1.8319	0.110	0.077
		O(OH)///Na	2.2582	0.343	0.470
	**2-EtOH**	H(OH)///O(OH)	1.7682	0.138	0.099
		O(OH)///H(OH)	1.9160	0.095	0.070
**GO**	**H_2O**	O(OH)///H	1.8921	0.094	0.068
		H(OH)///O	1.8692	0.110	0.085
	**EtOH**	$O(C_2O)$///H(OH)	2.0338	0.051	0.040
		$C(C_6)$///O(OH)	3.1263	0.024	0.028
		H(OH)///O(OH)	1.9511	0.085	0.063
		H(OH)///O(OH)	2.0206	0.074	0.058

Based on the obtained bond order data, it can be concluded that the strength of single hydrogen bonding interactions between the components of the composite material follows the sequence PEI>SA>GO (0.146>0.127>0.110 WBI, respectively) for water, and SA≥PEI>GO (0.138>0.130>0.085 WBI, respectively) for ethanol. The introduction of PEI and GO appears to lead to surface hydrophilization, potentially due to stronger hydrogen bonding interactions with components in the case of PEI and the presence of a greater

number of functional oxygen-containing groups in GO (confirmed by the contact angle data, Table 2).

A similar analysis was conducted for the coordination sodium clusters with water and ethanol (Figures S1 a, b). The interaction length, the ratio of interaction length to the sum of van der Waals radii (R) [93], and WBI values are presented in Table S5.

Table S5. The interaction length, the ratio of interaction length to the sum of van der Waals radii and WBI values.

B3LYP/6-311++G**				
**Associate**	**Interaction**	**d, Å**	**R**	**WBI**
SA···H_2O	Na///O(H_2O)	2.26332	0.60	0.344
	Na///O(CO_2)	2.35851	0.62	0.273
	Na///O(ring)	2.27298	0.60	0.289
	Na///O(OH)	2.48820	0.66	0.218
SA···EtOH	Na///O(EtOH)	2.25818	0.60	0.343
	Na///O(CO_2)	2.35369	0.62	0.273
	Na///O(ring)	2.27313	0.60	0.285
	Na///O(OH)	2.49292	0.66	0.218
SA···PEI	Na///N(PEI)	2.43362	0.64	0.340
	Na///O(CO_2)	2.23443	0.59	0.356
	Na///O(ring)	2.48776	0.66	0.180
	Na///O(OH)	2.30205	0.61	0.298

In the case of sodium clusters, the presence of critical points and bond paths between sodium and nitrogen atom from PEI or oxygen atoms from water/EtOH and SA indicates the existence of interactions. The formation of weak sodium complexes with ligands has been previously confirmed in the work [94]. In the current investigation, WBI calculated for structures range from 0.2 to 0.3, which suggests a borderline nature between noncovalent interactions and dative bonding [60, 61]. Additionally, significantly smaller R values less than 1 may indicate attractive nature of observed interactions [61]. This hypothesis was supported by the NMR spectra of ^{23}Na nuclei for GA cross-linked membranes (Figure 13), which showed an unresolved peak. The formation of additional peak for GA cross-linked membranes may be explained by the inclusion of alcohol and

water molecules into the inner sphere of the sodium ion, leading to formation new clusters and a signal in a weaker field [95].

The QTAIM method was utilized for systems presented in Table S3 to verify the noncovalent nature and existence of the observed interactions. In order to visualize weak interaction regions and identify the specific types of interactions, a color bar (Figure S3) indicating the value of sign (λ_2)ϱ was applied to the reduced density gradient isosurfaces (RDG). By utilizing this approach, areas of weak interaction can be visually identified, excluding the regions associated with covalent bonds. The resulting visualization includes bond critical points (CP) and noncovalent interaction plot (NCIplot) surfaces, which are specifically designed to characterize intermolecular interactions (Figure S4).

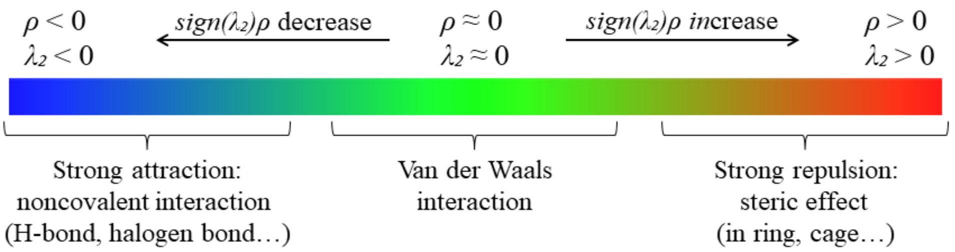

Figure S3. Discriminating color bar of weak interaction.

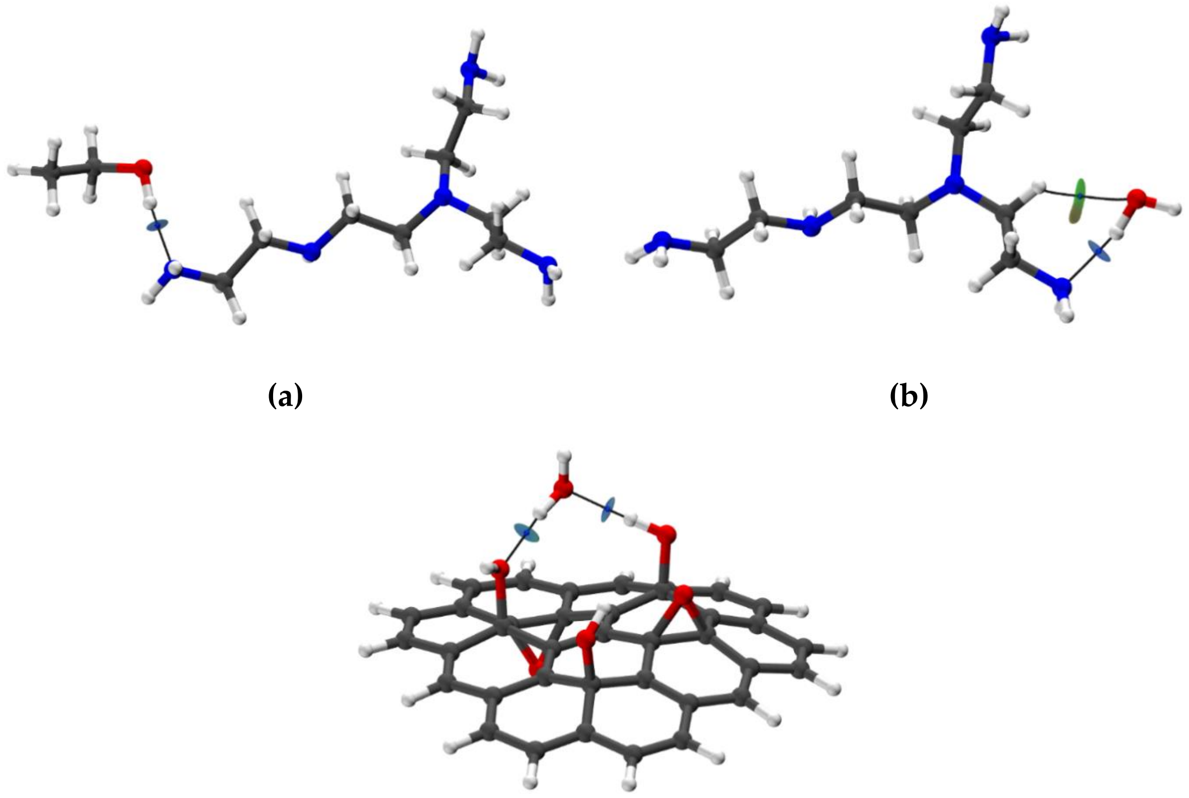

(a) **(b)**

(c)

Figure S4. QTAIM distribution of bond CPs (blue spheres) and bond paths for the associates **(a)** 2-PEI···EtOH, **(b)** PEI···H_2O, **(c)** GO···H_2O. The NCIplot index isosurfaces are

also represented using an isovalue of 0.5 ($e^{1/3}$ bohr)$^{-1}$, the color range is −0.04 e/bohr^3 ≤ sign(λ2)ϱ ≤ 0.02 e/bohr^3. CPs and NCIplot surfaces for characterization of intermolecular interactions are presented for clarity.

The analyses revealed the presence of bond critical points (BCPs) (3, −1), indicating the occurrence of noncovalent interactions and their types (Figures S1, S2, S3 and S4). The evaluated QTAIM parameters at the BCPs affirm the noncovalent and attractive nature of these bonds, as evidenced by the small values of sign (λ_2)ϱ at the BCPs (the product of the sign of the second eigenvalue of the electron density Hessian and the electron density). Detailed QTAIM parameters at the BCPs can be found in Table S6.

Table S6. Electron density ϱ(r) (e/bohr^3), Laplacian ∇^2ϱ(r) (e/bohr^5), potential energy density V(r), Lagrangian kinetic energy G(r) (Hartree) at the bond CP (3, −1), corresponding to different noncovalent interactions. ^{a}E,^{b}E, ^{c}E, ^{d}E (kJ/mol) are values of interaction energy.

B3LYP/6-311++G**										
**Associate**		**Interaction**	***p(r)***	**G(r)**	**V(r)**	**∇^2ϱ(r)**	**^{a}E***	**^{b}E***	**^{c}E***	**^{d}E***
**PEI**	**H_2O**	N(NH_2)///H	0.006	0.005	−0.004	0.024	−0.6	2.7	7.1	6.1
		H(CH_2)///O	0.033	0.023	−0.024	0.088	6.6	23.7	26.2	16.7
	**EtOH**	N(NH_2)///H(OH)	0.030	0.021	−0.021	0.082	5.9	21.1	23.4	15.2
	**SA**	H(NH)///O(CO_2)	0.015	0.011	−0.009	0.050	1.9	9.7	12.4	8.9
		H(CH_2)///O(CO_2)	0.004	0.003	−0.002	0.014	−1.2	0.4	5.6	5.6
		H(CH_2)///O(CO_2)	0.008	0.005	−0.005	0.024	−0.3	3.0	7.6	6.1
**SA**	**1-H_2O**	O(CO_2)///H	0.035	0.032	−0.031	0.127	7.3	34.0	33.7	29.0
	**2-H_2O**	H(COH)///O(OH)	0.032	0.028	−0.027	0.113	6.6	29.5	29.7	24.0
		O(COH)///H(OH)	0.033	0.033	−0.029	0.121	6.8	36.1	31.5	26.8
	**1-EtOH**	O(CO_2)///H(OH)	0.033	0.029	−0.029	0.121	6.7	31.4	31.1	26.6
	**2-EtOH**	H(OH)///O(OH)	0.037	0.032	−0.033	0.125	7.8	34.5	35.2	28.0
		O(OH)///H(OH)	0.028	0.024	−0.022	0.100	5.3	24.6	24.8	19.9
**GO**	**H_2O**	O(OH)///H	0.027	0.023	−0.021	0.100	5.0	23.8	23.5	19.9
		H(OH)///O	0.030	0.025	−0.023	0.103	5.8	25.9	26.0	20.9
	**EtOH**	O(C_2O)///H(OH)	0.019	0.016	−0.014	0.076	2.8	16.2	16.8	13.7
		H(OH)///O(OH)	0.025	0.020	−0.018	0.087	4.4	20.3	20.9	16.2
		H(OH)///O(OH)	0.022	0.017	−0.015	0.074	3.6	16.5	17.8	13.2

* *^{a}E, ^{b}E, ^{c}E, ^{d}E values of interaction energy in case of correlation with a - electron density ρ(r), b - Lagrangian kinetic energy G(r), c - potential energy density V(r), and d - Laplacian ∇^2ρ(r)* [96].
